# The ecological roots of human susceptibility to social influence: a pre-registered study investigating the impact of early-life adversity

**DOI:** 10.1098/rsos.180454

**Published:** 2019-01-09

**Authors:** Pierre O. Jacquet, Lou Safra, Valentin Wyart, Nicolas Baumard, Coralie Chevallier

**Affiliations:** 1 Laboratoire de neurosciences cognitives, Département d’études cognitives, École normale supérieure, INSERM, PSL Research University, 75005 Paris, France; 2 Institut Jean Nicod, Département d’études cognitives, ENS, EHESS, CNRS, PSL Research University, 75005 Paris, France

**Keywords:** early-life environment, human social influence, inter-individual variability, cognition, behavioural ecology, computational modelling

## Abstract

There is considerable variability in the degree to which individuals rely on their peers to make decisions. Although theoretical models predict that environmental risks shift the cost–benefit trade-off associated with social information use, this idea has received little empirical support. Here we aim to test the effect of childhood environmental adversity on humans' susceptibility to follow others’ opinion in the context of a standard face evaluation task. Results collected in a pilot study involving 121 adult participants tested online showed that susceptibility to social influence and childhood environmental adversity are positively associated. Computational analyses further confirmed that this effect is not explained by the fact that participants exposed to early adversity produce noisier decisions overall but that they are indeed more likely to follow the group's opinion. To test the robustness of these findings, a pre-registered direct replication using an optimal sample size was run. The results obtained from 262 participants in the pre-registered study did not reveal a significant association between childhood adversity and task performance but the meta-analysis ran on both the pilot and the pre-registered study replicated the initial finding. This work provides experimental evidence for an association between individuals' past ecology and their susceptibility to social influence.

## 1. Background

Modern western societies take for granted that intellectual autonomy, creativity and originality are universally valued. ‘Free-thinkers’, ‘rebels’ or ‘subversive attitudes' are indeed positively valued and parents even encourage their children to have their own opinion and to ‘be leaders rather than followers’. But such independence is in fact not highly regarded in every society and at every time in history [1]. Pre-industrial Europe, for instance, emphasized the importance of conformity and traditionalism, individuals took pride in following the ‘ancients’, and parents taught their children to be obedient, to revere their elders and to abide by the majority [2–4]. Within societies, individuals also vary in the degree to which they rely on others' views to make decisions and form opinions [5–8]. Why is that the case? Why are some environments seemingly more conducive to individual exploration while other environments promote more social forms of information acquisition?

Relying on social information to make decisions allows individuals to benefit from solutions that have already been tried out by their conspecifics. However, this strategy also comes with the risk of missing out on new, and potentially better, solutions that might have been discovered by trial-and-error exploration [9,10]. In gregarious species, individuals are thus expected to constantly weigh the cost and benefits of social information use on the one hand, and individual exploration on the other [11–15]. However, these trade-offs should also be calibrated as a function of a number of recurrent environmental pressures that modulate the fitness costs of social information use and individual exploration [16], leading to consistent preferences for one or the other strategy [17]. For example, in an environment characterized by high predation risks, individuals should rely more on information displayed by their peers to make their decisions. In more favourable environments, they should instead be in a better position to accommodate the costs associated with a longer sampling of the environment [18,19]. This theoretical model has been mostly applied to foraging decisions in non-human animals (e.g. deciding among several food patches, which one is the most profitable) [9,11,20], but it could also prove particularly useful to account for humans’ social decisions. Indeed, deciding whether or not to interact with a new partner can be construed as a typical case of exploratory behaviour associated with both a risk (of being exploited) and potential benefits (if the collaboration is successful). As such, following others' social judgements or relying on one's own opinion should depend on the same external factors that govern foraging decisions in other animals. To our knowledge, however, this hypothesis has never been tested despite the centrality of social learning biases in the human species [21]. Here, we thus test whether environmental factors account for part of the variation in how much people rely on social information to produce judgements about strangers.

Converging evidence in humans suggests that environmental stress experienced early in life is caused by two fundamental dimensions—harshness and unpredictability—that drive the adaptive calibration of an individual's psychology towards a sensitivity for short-term instead of long-term benefits [22] in various domains such as health, reproduction, parenting, economic decision-making or cooperation [23]. Harshness refers to the rates at which external morbidity–mortality cause disability and death in a given population. Unpredictability refers to the rates at which harshness varies over time and space [24]. An important cue for harshness experienced during childhood is the degree of resource scarcity characterizing individuals' households and neighbourhoods. Indeed, greater resource scarcity correlates with virtually all forms of morbidity and mortality [25,26], indicating that people who experienced scarcity in their childhood also experienced a greater exposure to disease, disability and death. Several studies on childhood unpredictability indicate that frequent changes or inconsistencies during childhood have a long-term impact on people's psychology, i.e. household instability and inconsistency, frequent residential change, etc. [27–32].

The effects of harshness and unpredictability are mostly convergent but it has been suggested that unpredictability could have a greater impact than harshness [33–35]. Up to a certain point indeed, harshness imposes tractable morbidity and mortality threats, which can be somewhat buffered by the maintenance of long-term behavioural strategies (e.g. increased parental investment) that shield individuals from predictable fitness costs [24]. Unpredictability, on the other hand, merely increases the temporal variance of these risks, but in the absence of consistent cues to predict what the environment will look like in the future, stochastic conditions are likely to favour present-oriented behaviours [24].

This research allows us to make specific predictions about the effect of harshness and unpredictability on individuals' susceptibility to rely on social rather than individual information. First, we predict that perceived childhood harshness and unpredictability increase susceptibility to social influence. Second, we predict that childhood unpredictability has a greater impact on individuals’ behavioural strategy than childhood harshness. Finally, we predict that the effect of childhood adversity, i.e. the combination of harshness and unpredictability, is greater than the effect of each of its dimensions taken in isolation.

**Figure 1.**
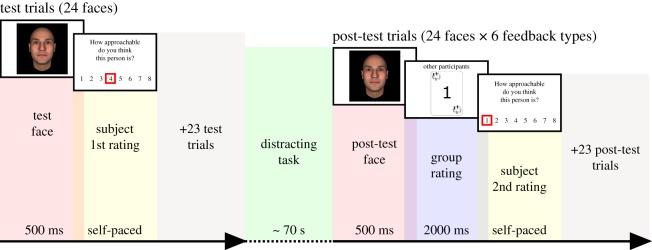
Experimental procedure. During test trials, participants rated a series of 24 computerized faces (randomized by participants) on approachability by clicking on one of eight possible values on the scale. After completing this first phase, participants performed a distracting task lasting approximately 70 s. Differences between the distracting task in the pilot and in the target study are detailed in the Material and methods section. Participants then completed the post-test, in which all the faces presented in the test phase are presented again for 500 ms. Each face is followed by the presentation of the rating provided by the simulated group of peers, for a duration of 2000 ms. Participants were then asked to provide a second rating for each face. In the present example, comparing the participant's test (first) and post-test (second) ratings indicates that she was influenced by the information displayed by the group.

To test these hypotheses, we adapted a well-validated face evaluation task [36–41] (figure 1), asking participants to rate unfamiliar faces on ‘approachability’ before and after seeing the rating of a simulated group of conspecifics. Social information consists in displaying the most frequent rating provided by a group of peers. This rating can deviate positively or negatively from participants' initial rating to a moderate or high degree (disagreement conditions), or not deviate at all (agreement condition). Behavioural inter-individual variability is analysed in light of perceived childhood environmental harshness and unpredictability independently and of childhood environmental adversity (harshness and unpredictability combined). A computational model of choice is also used to analyse the weight participants attribute to social information during post-test ratings.

## 2. Pilot study

### 2.1. Material and methods

#### 2.1.1. Participants

In order to assess sample size, we capitalized on an existing dataset that was initially collected online via the Mechanical Turk recruitment platform to test the interaction effect of childhood environment and disgust sensitivity on susceptibility to social influence. All phases of the study (the informed consent, the pilot task and questionnaires) were coded using Qualtrics (Qualtrics, Provo, UT, ©2016) and presented in a Web browser. The pilot experiment compared the influence exerted by social information on face approachability ratings in a condition where participants were primed with disgusting images and in another condition where they were primed with neutral images [42]. For our current purposes, we restricted our analysis to the neutral condition (a condition that is fairly similar to the one we aim to use in the target study). A target sample size of 140 US participants was chosen *a priori* and 143 participants completed the study. A series of quality checks was performed to ensure that each participant (i) had declared being 18 years old or more, (ii) had entered the correct verification code (generated by Qualtrics at the end of the procedure), (iii) did the task only once (by comparing the participants’ IP addresses and the GPS coordinates provided by Qualtrics) and (iv) had enough valid data in each experimental condition. A total of 125 participants (59 females, 66 males) fulfilled these criteria (mean age = 34 ± 9). All participants reported being naive to the purpose of the experiment, gave their written informed consent and received payment for their participation in accordance with the standards of Mechanical Turk. The experimental protocol was approved by the local Ethical Committee (Conseil d’évaluation éthique pour les recherches en santé—CERES n°201659) and is in accordance with the Declaration of Helsinki (World Medical Association, 2008).

#### 2.1.2. Stimuli and procedure

The stimuli consisted of 24 emotionally neutral faces generated using FaceGen Modeller 3.5 (Singular Inversions, 2007) according to the methods developed by Oosterhof & Todorov [43]. The experimental procedure was adapted from [36]. It lasted 16 min (±7 min) on average and was divided into three phases.

In a first phase, participants watched a series of 24 neutral faces presented for 500 ms, a duration that has been shown to minimize participants' ability to encode the identity of faces but that provides enough time to consistently estimate social traits such as trustworthiness [43–45]. After the presentation of each face, a numerical scale appeared to assess approachability. The scale remained on the screen until participants selected a value between 1 and 8 (figure 1). Immediately after the test phase, participants performed a distracting task where they were asked to look at six neutral images each presented three times for 3 s [42], and to judge how disgusting they were on an 8-point scale (1 = not disgusting at all, 8 = very disgusting). In a third phase, participants were asked to rate the same faces again (in a random order) and they were informed that they would see other MTurkers’ modal rating after seeing each face. This social feedback was represented on a card featuring a social cue in its angles (figure 1). Unbeknownst to the participants, the group rating was bogus and generated online by means of a simple algorithm. Note that previous studies using a similar procedure have shown that participants are convinced that the group ratings are provided by real individuals [36–41].

The social feedback included 12 disagreement trials equally split between four possible outcomes: the bogus group rating was either higher than the participant's initial rating (positive disagreement) or lower (negative disagreement); and the deviation was either moderate (+2/−2 points deviation) or strong (+3/−3 points deviation). In sum, disagreement trials varied in terms of disagreement valence (positive versus negative) and disagreement strength (moderate versus strong), following a 2 × 2 design. Two control conditions were also introduced: in agreement trials (six trials), the group rating matched the participant's initial rating; in no feedback trials (six trials), no feedback was displayed. Agreement and no feedback trials allowed us to control that participants selectively adjusted their ratings based on the type of feedback they were exposed to, and to check that childhood environment did not bias mean rating change in a negative or positive direction in situations where participants had no particular reason to do so.

#### 2.1.3. Assessing susceptibility to social influence

We first examined the extent to which participants *adjusted* their ratings by looking at the mean difference between test and post-test ratings. Mean rating change was computed in no feedback trials, in agreement trials, and in all disagreement trials (strong positive disagreement, moderate positive disagreement, strong negative disagreement, moderate negative disagreement). Positive and negative mean rating change, respectively, indicate that participants increase and decrease their approachability ratings in the post-test.

Social influence was defined as cases where participants *aligned* their post-test ratings in the direction of the information provided by the group or, said differently, adjusted their ratings *in line* with the group (e.g. a positive mean rating change in positive disagreement trials and a negative mean rating change in negative disagreement trials). In order to obtain a social alignment score across disagreement conditions, we reversed the sign of the mean rating change obtained in negative disagreement trials: a positive social alignment score thus indicates that participants adjusted their ratings towards the group in both positive and negative disagreement trials and a negative score indicates that participants adjusted their ratings away from the group. Thus, the greater the social alignment score, the greater the participant's susceptibility to social influence.

#### 2.1.4. Assessing early-life environment

Exposure to adverse environments, including unpredictability and harshness, was assessed using established questionnaires [46–50]. Unpredictability was assessed following the methods developed by Mittal and colleagues [32]. Participants first read the following instructions: ‘Think back to your life when you were younger than ten. This time includes preschool, kindergarten, and the first few years of elementary school.’ They were then asked to say how much they agreed with the following three statements: ‘When I was younger than 10… : (a) things were often chaotic in my house, (b) people often moved in and out of my house on a pretty random basis, and (c) I had a hard time knowing what my parent(s) or other people in my house were going to say or do from day-to-day.’ Responses to these three items were made on a 7-point scale ranging from 1: strongly disagree, to 7: strongly agree. Item scores were averaged and *z*-scored to provide a single score of childhood unpredictability [32]. Thus, the higher the score, the less predictable the participant perceived her childhood environment.

We used perceived childhood scarcity (until the age of ten) as a proxy for early-life environmental harshness [50–52] and relied on an established three-item questionnaire [32,46–49]: (a) ‘My family usually had enough money for things when I was growing up’, (b) ‘I grew up in a relatively wealthy neighbourhood’, (c) ‘I felt relatively wealthy compared to the other kids in my school’. Responses were made on a 7-point scale ranging from 1: strongly disagree, to 7: strongly agree. Item scores were then reversed, averaged and z-scored to provide a single score of childhood harshness [50]. Thus, the higher the score, the harsher the participant perceived her childhood environment.

Finally, the *z*-transformed unpredictability and harshness scores were summed in order to obtain a single score of childhood adversity. We used this adversity score to test the synergistic effect of childhood environmental harshness and unpredictability on susceptibility to social influence.

#### 2.1.5. Data quality check and data cleaning

A visual inspection of our outcome measure using *P–P* plots suggested that the distribution of mean rating change observed in each type of group disagreement approximately matched the theoretical normal distribution (see also electronic supplementary material, figure S1 for frequency plots). This was also supported by acceptable ranges of skewness and kurtosis parameters (*skewness*: −0.14–0.31; *kurtosis*: −0.19–0.28), and by results of Shapiro–Wilk's *W* tests (all *W*s > 0.97, all *p*s > 0.052). Finally, a Levene's test of the homogeneity of variances was conducted to verify the assumptions of the general linear model. The test suggested that the mean rating change variance for the four types of group disagreement did not significantly differ, hence fulfilling this assumption (*p* = 0.15). An inter-item reliability analysis performed on the item scores collected on the 125 pilot study participants showed that childhood unpredictability and harshness questionnaires had a satisfactory internal consistency (*Cronbach's alpha*: *unpredictability* = 0.79, *harshness* = 0.77). The two scales were moderately correlated (*r* = 0.37). Two participants were outliers on the social alignment score, and two were outliers on the childhood unpredictability score (using the 1.5 times interquartile range criterion). These four participants were discarded from subsequent analyses, and the final sample thus included 121 individuals (hence representing a total loss of 15%).

### 2.2. Statistical and computational analyses of behavioural data

All analyses were performed using Matlab version R2014b and R. Participants' performances were analysed in a series of *t*-tests and linear mixed models with the *lme* function of the *nlme* R package [53]. All models used a maximum-likelihood fitting method and had random intercepts. Bayes factors (*BF*_10_) with default Jeffreys–Zellner–Siow (JZS) priors were further calculated using the *lmBF* function to compare the predictive power of models [54]. Values superior to 1 indicate greater evidence for the alternative model, while values inferior to zero indicate greater evidence for the reference model. Comparing models using Bayes factors allowed us to determine whether childhood harshness, unpredictability and adversity have an effect on social alignment scores and, if they do, which environmental variable has the greatest effect.

#### 2.2.1. Positive control: effect of trial type on mean rating change

As a first step, a series of *t*-tests (*t*-tests for a single mean, *t*-tests for dependent samples) was run on participants’ mean rating change in each trial type in order to check that disagreement trials elicited more change in participants' ratings than control trials.

#### 2.2.2. Effect of disagreement valence and strength on social alignment scores

The main effect of disagreement valence (positive versus negative disagreement) and disagreement strength (moderate versus strong disagreement), as well as their interaction effect on social alignment scores, were analysed using a linear mixed model taking disagreement valence and disagreement strength as within-subject fixed-effect factors, and participants’ ID as a random factor. This model served as a baseline for model comparison analyses.

#### 2.2.3. Effect of childhood environment on social alignment scores

We then investigated the contribution of childhood environment on participants' social alignment scores by enriching the baseline model with childhood harshness, childhood unpredictability and childhood adversity successively. Each predictor was either included as a main effect (alternative type 1 model) or as an interaction term with disagreement valence and disagreement strength (alternative type 2 model). A first-order Bayes factor was calculated for each model and provided an indication of the predictive power of each model relative to a null model including the intercept only. A second-order Bayes factor was then computed to compare the predictive power of alternative models with the baseline model taken as the reference. Alternative models associated with a Bayes factor greater than that of the baseline model provide evidence for an effect of childhood environment on social alignment scores. Finally, we calculated a third-order Bayes factor to compare the best fitting alternative model found for childhood unpredictability and for childhood adversity with the best fitting alternative model found for childhood harshness. A greater Bayes factor suggests greater evidence for an effect of unpredictability or adversity on social alignment scores. A similar comparison was done for unpredictability and adversity.

#### 2.2.4. Effect of childhood environment on mean rating change in the control conditions

A similar analytic strategy was used to study the effect of childhood environment on mean rating change following agreement and no feedback trials. We first generated a baseline linear mixed model taking mean rating change as the dependent variable, a two-level within-subject fixed-effect factor including the two control conditions, and participants’ ID as a random factor. We then compared this model with alternative models including the childhood environmental variable of interest either as a main effect (alternative type 1 model) or as an interaction term (alternative type 2 model). A first-order Bayes factor was calculated for each model, providing an indication of the predictive power of each model relative to a null model including the intercept only. A greater Bayes factor for the baseline model suggests that there is evidence in favour of an effect of childhood environmental variables on the mean rating change in the control conditions.

#### 2.2.5. Computational model description and fitting

The fitted computational model hypothesizes that the decision to adjust a rating after the integration of a social feedback is formed on the basis of a comparison between the face presented in post-test trials and the type of social feedback it is paired with. The model consists of two free parameters, fitted to each participant's answers: (1) a social influence parameter *δ* corresponding to the weight attributed by the participant to the group rating with respect to her own initial rating, and which determined the adjustment of the post-test rating, here measured as the signed fraction of the distance (i.e. disagreement) between the initial rating and the subsequent group rating (positive for adjustments in line with the group's rating, negative for adjustments away from the group's rating), and (2) an internal noise magnitude parameter *σ* corresponding to the standard deviation of the post-test rating. The mean rating in post-test trials *μ* thus corresponds to a linear combination between the initial rating *x*_ini_ and the group rating *x*_group_ following:

$$\mu =x_{\mathrm{ini}}\cdot (1-\delta )+x_{\mathrm{group}}\cdot \delta .$$

The probability of choosing the discrete rating *x* in post-test trials can be computed using the following equation:

$$p(x)=\Phi \left(x+\frac{1}{2},\mu ,\sigma \right)-\Phi \left(x-\frac{1}{2},\mu ,\sigma \right),$$

where *Φ*(.) is the cumulative normal density function.

We obtained maximum-likelihood estimates of the two parameters *δ* and *σ* separately for each participant using gradient descent of the negative model likelihood using the ‘interior-point’ algorithm of the *fmincon* routine implemented in Matlab. We derived model predictions in terms of social alignment scores for all measures that were made directly from participants' behaviour, as a means to test the adequacy of the model.

### 2.3. Results

#### 2.3.1. Effect of trial type on mean rating change

**Figure 2.**
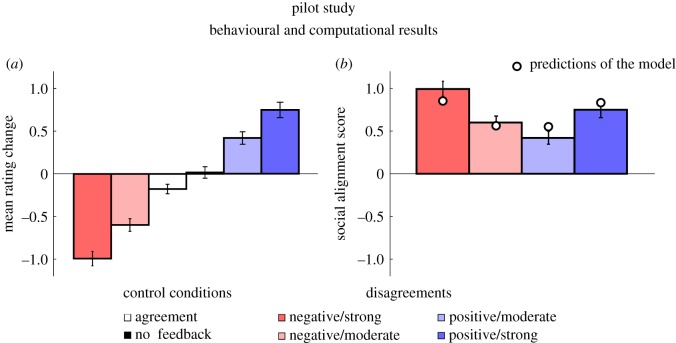
Pilot study: behavioural and computational results (*N* = 121). (*a*) Effect of feedback type on mean rating change (±s.e.m.). A positive or negative mean rating change (*y*-axis) indicates that participants increased or decreased their approachability ratings in post-test trials, and a near-0 mean rating change indicates that participants' ratings did not change in post-test trials. (*b*) Effect of group disagreement on social alignment scores (±s.e.m.). A positive social alignment score (*y*-axis) indicates that participants adjusted their approachability ratings in line with the group, a negative social alignment score indicates that they adjusted their approachability ratings away from the group, and a near-0 score indicates that participants’ rating changes were not biased in any direction. The discs represent the predictions of the computational model for each type of disagreement (see Material and methods for details).

A first look at the data indicates that participants were more likely to change their ratings following a disagreement with the group than in the control conditions (figure 2*a*). Indeed, the mean rating change differed from zero in all types of disagreement trials (−2 point disagreement: *M* = −0.60, s.e.m. = 0.07; −3 point disagreement: *M* = −0.99, s.e.m. = 0.08; +2 point disagreement: *M* = 0.42, s.e.m. = 0.07; +3 point disagreement: *M* = 0.75, s.e.m. = 0.09). In agreement trials, the mean rating change also differed from zero, though to a smaller extent (*M* = −0.18, s.e.m. = 0.06; *t* = −3.2, *p* < 0.002). In the no feedback trials, however, the mean rating change did not differ from zero (*M* = 0.02, s.e.m. = 0.07; *t* = 23.7, *p* = 0.82). Importantly, the mean rating change obtained in each type of disagreement trials differed from the mean rating change obtained in agreement trials (all *t*s > 6.85, all *p*s < 0.001) and in no feedback trials (all *t*s > .05, all *p*s < 0.001).

#### 2.3.2. Effect of disagreement valence and strength on social alignment scores

The social alignment score was obtained by reversing the sign of the mean rating change obtained in negative disagreement trials (see §2.1.3. for details). Results of the baseline linear mixed model on social alignment scores showed that, on average, participants’ rating alignment was greater when exposed to strong disagreement than to moderate disagreement (*β* = 0.33 ± 0.10, *t*_360_ = 3.15, *p* = 0.002). Neither disagreement valence (*t*_360_ = 1.72, *p* = 0.09) nor its interaction with disagreement strength (*t*_360_ = 0.44, *p* = 0.66) had an effect on social alignment scores (figure 2*b*).

#### 2.3.3. Effect of childhood environment on social alignment scores

**Figure 3.**
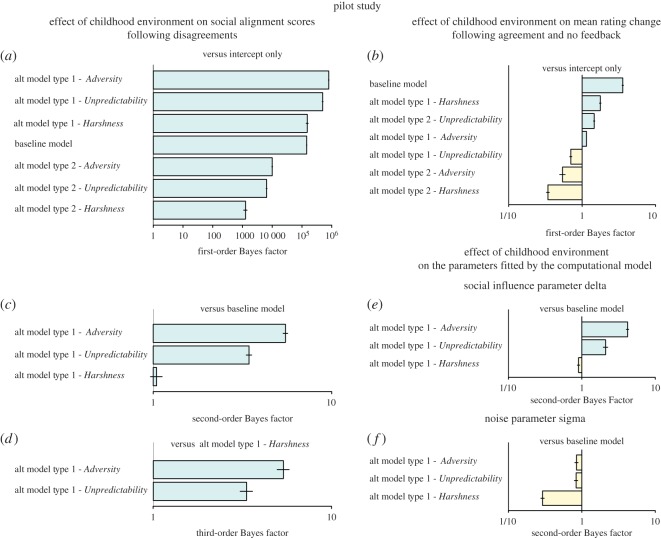
Analyses of the pilot dataset. Bayesian analyses of models with and without environmental variables as predictors of social alignment score in disagreement trials (*a*–*c*), of mean rating change in agreement trials (*d*), and of the parameters delta (*e*) and sigma (*f*) fitted by our computational model. The baseline model does not include any environmental variable; alternative models include each environmental variable as a main effect (type 1) or as a term interacting with disagreement valence and disagreement strength (type 2) (*a*–*c*,*e*,*f*) or control conditions (*d*). First-order Bayes factors compare the baseline model and alternative type 1 and 2 models with a null model including the intercept only. Second-order Bayes factors compare alternative type 1 and 2 models with the baseline model. Third-order Bayes factors compare the childhood adversity and unpredictability models with the childhood harshness model. A Bayes factor less than 1 indicates evidence in favour of the reference model.

A general overview of the first-order Bayes factor indicates that models with childhood environmental variables as independent fixed-effects predicted the observed data better than other models (figure 3*a*), including the baseline model and alternative type 2 models.

The second-order Bayes factor further revealed that including childhood unpredictability or adversity improved the likelihood of the model by, respectively, 3.5 and 5.5 times (*Unpredictability* versus *Baseline*: *BF*_10_ = 3.45 ± 3.42%; *Adversity* versus *Baseline*: *BF*_10_ = 5.51 ± 2.94%). Positive evidence in favour of the alternative model including childhood harshness was more equivocal (*Harshness* versus *Baseline*: *BF*_10_ = 1.04 ± 7.47) (figure 3*b*). Together, these results suggest that childhood environment has a general effect on social alignment scores regardless of disagreement valence and disagreement strength. This was further supported by the mixed effect models that showed that increased childhood adversity was associated with increased social alignment scores (*Harshness*: *β* = 0.10 ± 0.05, *t*_119_ = 2.06, *p* = 0.04; *Unpredictability*: *β* = 0.13 ± 0.05, *t*_119_ = 2.72, *p* = 0.008; *Adversity*: *β* = 0.09 ± 0.03, *t*_119_ = 2.93, *p* = 0.004) (see electronic supplementary material, figure S4). Note that all type 1 models including environmental variables as main predictors performed better than similar models that included age (*Harshness* versus *Age*: *BF*_10_ = 3.75 ± 7.28%; *Unpredictability* versus *Age*: *BF*_10_ = 11.92 ± 3.76%; *Adversity* versus *Age*: *BF*_10_ = 22.5 ± 5.08%) or gender as predictors (*Harshness* versus *Gender*: *BF*_10_ = 1.79 ± 7.12%; *Unpredictability* versus *Gender*: *BF*_10_ = 5.69 ± 3.45%; *Adversity* versus *Gender*: *BF*_10_ = 10.745.51 ± 4.86%). Models' parameters confirmed that neither age, nor gender, had a significant effect on social alignment scores (*Age*: *β* = −0.006 ± 0.006, *t*_119_ = −1.04, *p* = 0.30; *Gender*: *β* = −0.18 ± 0.10, *t*_119_ = −1.79, *p* = 0.08).

As a final step, we compared the explanatory power of models involving childhood environmental variables and found positive evidence in favour of the childhood unpredictability model relative to the childhood harshness model *(Unpredictability* versus *Harshness*: *BF*_10_ = 3.30 ± 7.8%). We also found that the model including childhood adversity—the combination of harshness and unpredictability—yielded a greater probabilistic prediction for the observed data than the model including childhood harshness (*Adversity* versus *Harshness*: *BF*_10_ = 5.23 ± 7.6%) and, more equivocally, than the model including childhood unpredictability (*Adversity* versus *Unpredictability: BF*_10_ = 1.60 ± 3.7) (figure 3*c*).

#### 2.3.4. Effect of childhood environment on mean rating change in the control conditions

In line with our predictions, Bayesian analyses showed no evidence that childhood environment affects mean rating change in the agreement and the no feedback trials. None of the models including childhood environmental variables as predictors outperformed the baseline model (figure 3*d*). This was further confirmed by examining the coefficients of the main effect of childhood environmental variables estimated by the alternative linear mixed-effects models (*t* range = −0.43–−1.70, *p* range = 0.09–0.86).

To summarize, our pilot study allowed us to test the effect of harshness and unpredictability on individuals’ susceptibility to rely on social rather than individual information. In line with our predictions, we found that (1) perceived childhood harshness and unpredictability increased susceptibility to social influence; (2) childhood unpredictability had a greater impact on individuals' susceptibility to social influence than childhood harshness; (3) the effect of childhood adversity (harshness and unpredictability combined) is greater than the effect of each of its dimensions taken in isolation. Several possible mechanisms might account for these results. One is that individuals who grew up in more adverse environments rely more on social information, another possible mechanism is that the accumulation of stress caused by early adversity has a negative impact on brain functioning and higher-level cognition (e.g. working memory, decision-making, etc.), which then impacts performance in the task [29–33]. In what follows, we turn to computational models of choice to disentangle these two possibilities.

#### 2.3.5. Computational analyses of behavioural data

We fitted participants’ behaviour using a canonical model of choice that hypothesizes that the decision to adjust a rating following social feedback is based on a comparison between the face and the group rating it is paired with (see Material and methods). The model consists of two free parameters—a social influence parameter *δ* and an internal noise magnitude parameter *σ*—fitted to each participant's responses. We obtained maximum-likelihood estimates of these two parameters from each participant's behaviour (see Materials and methods) and then compared the predictions made by the model with the participants' social alignment scores. All the effects were predicted by the computational model (figure 2*b*, see also electronic supplementary material, §1.1.5. and figure S5 for detailed analyses).

We therefore conducted an analysis comparing a baseline model (without childhood environmental variables) and alternative type 1 models (with childhood environmental variables) with a null model (including the intercept only), each of them taking *δ* or *σ* as the dependent variable separately. The pattern of results extracted from the Bayesian analysis of observed social alignment scores was replicated with the fitted social influence parameter *δ*. A second-order Bayes factor showed that the model including childhood unpredictability and adversity was, respectively, two times and four times more likely than the baseline model (*Unpredictability* versus *Baseline*: *BF*_10_ = 2.11 ± 6.83%; *Adversity* versus *Baseline*: *BF*_10_ = 4.19 ± 3.48%) (figure 3*e*). Again, all models including environmental variables as main predictors performed better than similar models including age (*Harshness* versus *Age*: *BF*_10_ = 3.75 ± 7.28%; *Unpredictability* versus *Age*: *BF*_10_ = 11.92 ± 3.76%; *Adversity* versus *Age*: *BF*_10_ = 22.5 ± 5.08%) or gender (*Harshness* versus *Gender*: *BF*_10_ = 1.79 ± 7.12%; *Unpredictability* versus *Gender*: *BF*_10_ = 5.69 ± 3.45%; *Adversity* versus *Gender*: *BF*_10_ = 10.745.51 ± 4.86%). Models’ parameters confirmed that neither age, nor gender, had a significant effect on social alignment scores (*Age*: *β* = −0.002 ± 0.002, *t*_119_ = −0.79, *p* = 0.42; *Gender*: *β* = −0.08 ± 0.04, *t*_119_ = −1.86, *p* = 0.07).

When the noise parameter *σ* was taken as the dependent variable, however, second-order Bayes factors showed no improvement of the model's predictive power after the inclusion of childhood environmental variables in the model, when compared to the baseline model (figure 3*f*). While the noise parameter *σ* remained relatively unaffected overall (*Harshness*: *β* = 0.06 ± 0.05, *t*_119_ = 1.24, *p* = 0.22; *Unpredictability*: *β* = 0.10 ± 0.05, *t*_119_ = 2.01, *p* = 0.05; *Adversity*: *β* = 0.06 ± 0.03, *t*_119_ = 1.98, *p* = 0.05), the social influence parameter *δ* increased as long as participants' childhood environment was perceived as more adverse (i.e. the less predictable and the harsher) (*Harshness*: *β* = 0.04 ± 0.02, *t*_119_ = 2.07, *p* = 0.04; *Unpredictability*: *β* = 0.05 ± 0.02, *t*_119_ = 2.50, *p* = 0.01; *Adversity*: *β* = 0.03 ± 0.01, *t*_119_ = 2.80, *p* = 0.006). These results therefore suggest that the positive effect of childhood environment on susceptibility to social influence is mediated by an increased sensitivity to social feedbacks and is not an indirect by-product of noisy evidence accumulation or decision processes.

## 3. Pre-registered study

The goal of the pre-registered study is to test the strength and reliability of the effect of childhood environment on susceptibility to social influence by replicating the pilot study with heightened statistical power. The stimuli and procedure in the pre-registered study were identical to the pilot study with the exception of two minor differences. First, we modified the distracting task separating the test and the post-test phase. The rationale was that even though the images used in the pilot studies were judged as neutral (8-point scale: *M* = 2.99, s.d. = 1.22), their content might nonetheless interfere with the task. Therefore, instead of judging neutral images on the disgust dimension, participants were engaged in a fully neutral task where they compared the surfaces of two squares, i.e. a grey square and a black square (see electronic supplementary material, figure S6). Second, we assessed participants’ current environment by adapting the childhood harshness and unpredictability questionnaires used in the pilot study [46–50] (see the electronic supplementary material, §2.1.4.). The analysis plan was strictly identical to the one used in the pilot study. Full descriptions of the materials and methods, the analytic strategy, the power analyses and sample size estimations are provided in the electronic supplementary material, §2.3.

The pre-registered protocol, unchanged from the point of Stage 1 acceptance, can be found in the Open Science Framework at https://osf.io/nvzdb/.

### 3.1. Results

#### 3.1.1. Participants included in the pre-registered protocol

We recruited 340 participants via Amazon Mechanical Turk (342 participants were finally able to complete the experiment). Twenty-one MTurkers with IP addresses located outside the US were automatically excluded. The sample was therefore composed of 321 US participants. Forty-six of them did not fulfil the quality checks, and 13 were outliers (using the 1.5 times interquartile range criterion) on social alignment scores averaged across all types of disagreement (no participant was an outlier on harshness or unpredictability scores). These 59 participants were excluded from the final analyses, representing a total loss of approximately 18% (a value close to the one we predicted, see the last paragraph of the electronic supplementary material, §2.3). We were thus left with a final sample of 262 individuals (154 females, 108 males; mean age = 38 ± 12).

#### 3.1.2. Effect of trial type on mean rating change

**Figure 4.**
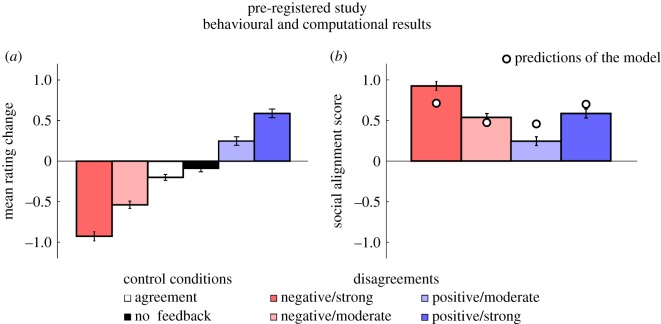
Pre-registered study: behavioural and computational results (*N* = 262). (*a*) Effect of feedback type on mean rating change (±s.e.m.). A positive or negative mean rating change (*y*-axis) indicates that participants increased or decreased their average approachability ratings in post-test trials, and a near-0 mean rating change indicates that participants' ratings did not change in post-test trials. (*b*) Effect of group disagreement on social alignment scores (±s.e.m.). A positive social alignment score (*y*-axis) indicates that participants adjusted their approachability ratings in line with the group, a negative social alignment score indicates that they adjusted their approachability ratings away from the group, and a near-0 score indicates that participants’ rating changes were not biased in any direction. The discs represent the predictions of the computational model for each type of disagreement (see Material and methods for details).

As in the pilot study, the mean rating change differed from zero in all types of disagreement trials (−2 point disagreement: *M* = −0.54, s.e.m. = 0.78; −3 point disagreement: *M* = −0.93, s.e.m. = 0.85; +2 point disagreement: *M* = 0.24, s.e.m. = 0.89; +3 point disagreement: *M* = 0.58, s.e.m. = 0.92; all *t*s > 4.31, all *p*s < 0.001). The mean rating change was statistically different from zero in the agreement condition (*M* = −0.20, s.e.m. = 0.62; *t* > 5.25, *p* < 0.001), and at the significance threshold in the no feedback condition (*M* = −0.09, s.e.m. = 0.73; *t* = 1.97, *p* = 0.05). In addition, the mean rating change obtained in each type of disagreement trials differed from the mean rating change obtained in agreement trials (all *t*s > 6.53, all *p*s < 0.001) and in no feedback trials (all *t*s > 4.58, all *p*s < 0.001). Participants were therefore more likely to change their ratings following a disagreement with the group than in the control conditions, a result that matched the one obtained in the pilot study (figure 4*a*).

#### 3.1.3. Effect of disagreement valence and strength on social alignment scores

The social alignment score was obtained by reversing the sign of the mean rating change obtained in negative disagreement trials (see §2.1.3. for details). As in the pilot study, results of the baseline linear mixed model on social alignment scores showed that participants' susceptibility to social alignment was greater when exposed to strong disagreement than to moderate disagreement (*β* = 0.39 ± 0.07, *t*_783_ = 5.42, *p* < 0.001). Unlike in the pilot study, however, disagreement valence had a main effect. The social alignment score was indeed greater in negative disagreement than in positive disagreement (*β* = 0.29 ± 0.07, *t*_783_ = 4.11, *p* < 0.001). Valence and strength of disagreement did not interact (*t*_783_ = 0.45, *p* = 0.65) (figure 4*b*).

#### 3.1.4. Effect of childhood environment on social alignment scores

The pilot study revealed a positive influence of childhood unpredictability and adversity on the social alignment score. We did not replicate this result in the pre-registered study. A general overview of the first-order Bayes factor did show that type 1 models with environmental variables as main effects were better at predicting the data than type 2 models with environmental variables as interaction terms. However, none of these type 1 models outperformed the fit of the baseline model (all *BF*_10_s < 0.18) and models parameters indicated that childhood harshness, unpredictability or adversity had no significant effect on social alignment scores (*Childhood Harshness*: *β* = 0.18 ± 0.03, *t*_260_ = 0.59, *p* = 0.55; *Childhood Unpredictability*: *β* = 0.037 ± 0.03, *t*_260_ = 1.21, *p* = 0/22; *Childhood Adversity*: *β* = 0.02 ± 0.02, *t*_260_ = 1.08, *p* = 0.28). Note that the replacement of childhood environmental variables by current environmental variables did not yield better results (all *BF*_10_s < 0.29, all *β*s < 0.05, all *ts* < 1.62, all *p*s > 0.10).

#### 3.1.5. Effect of childhood environment on mean rating change in the control conditions

In line with the pilot study, childhood environmental variables had no effect on control trials (agreement and no feedback trials). Bayesian analyses showed that models with environmental variables as predictors did not outperform the baseline model (all *BF*_10_s < 0.26). This was confirmed by examining the coefficients of the main effect of environmental variables estimated by type 1 (*t* range = −0.32–1.34, *p* range = 0.18–0.75) and type 2 models (*t* range = 0.05–0.52, *p* range = 0.61–0.96).

Similar results were obtained after replacing childhood environmental variables with current environmental variables (all *BF*_10_s < 0.26, all *β*s < 0.11, all *t*s < 2.06, all *p*s > 0.04). The only difference was that the type 2 model including current unpredictability as an interaction term provided more evidence than the baseline model (*BF*_10_s = 1.68). This was explained by the fact that current unpredictability scores and mean rating change were associated in agreement trials but not in no feedback trials (*β* = −0.10 ± 0.03, *t*_260_ = −2.99, *p* = 0.003).

#### 3.1.6. Computational analyses of behavioural data

Logically, the analyses conducted on the social influence parameter *δ* measured by our computational model also showed that the inclusion of childhood environmental variables did not increase the fit of the data relative to the baseline model (all *BF*_10_s < 0.15). More specifically, the parameters of type 1 models confirmed the absence of relation between participants’ childhood environment and the social influence parameter *δ* (*Childhood Harshness*: *β* = 0.007 ± 0.01, *t*_260_ = 0.55, *p* = 0.58; *Childhood Unpredictability*: *β* = 0.02 ± 0.01, *t*_260_ = 1.21, *p* = 0.23; *Childhood Adversity*: *β* = 0.008 ± 0.01, *t*_260_ = 1.05, *p* = 0.29).

The analyses of the noise parameter *σ* taken as the dependent variable showed that the inclusion of childhood environmental variables as main effects did not improve the model's fit, compared to the baseline model (all *BF*_10_s < 0.36). The noise parameter *σ* remained unaffected (*Childhood Harshness*: *β* = 0.006 ± 0.02, *t*_260_ = 0.31, *p* = 0.76; *Childhood Unpredictability*: *β* = 0.03 ± 0.02, *t*_260_ = 1.70, *p* = 0.09; *Childhood Adversity*: *β* = 0.01 ± 0.01, *t*_260_ = 1.21, *p* = 0.23).

Note that current environmental variables were not found to affect the social influence parameter *δ* (all *BF*_10_s < 0.29, all *β*s < 0.02, all *t*s < 1.59, all *p*s > 0.11), nor the noise parameter *σ* (all *BF*_10_s < 0.47, all *β*s < 0.02, all *t*s < 1.82, all *p*s > 0.07).

### 3.2. Unregistered analyses

#### 3.2.1. Meta-analysis

Recent methodological practices state that determining a successful replication can no longer be based solely on whether or not each single study achieved significance [55]. Pooling into a single estimate the individual effects sizes of the completed studies as well as quantifying their heterogeneity is generally more trustworthy, notably because the outputs are based on far more data than each individual study. As demonstrated by Braver *et al.* [55], it comes out that a meta-analysis on a replication attempt that does not reach significance might nonetheless provide more, not less, evidence that effect is real. Following recent recommendations, we therefore ran a meta-analysis of the pilot and the pre-registered studies using a fixed-effects meta-analysis model implemented in the ‘metafor’ R package [56]. Using a fixed-effects model indeed allows us to test how large the average true effect is in the two studies included in the meta-analysis.

**Figure 5.**
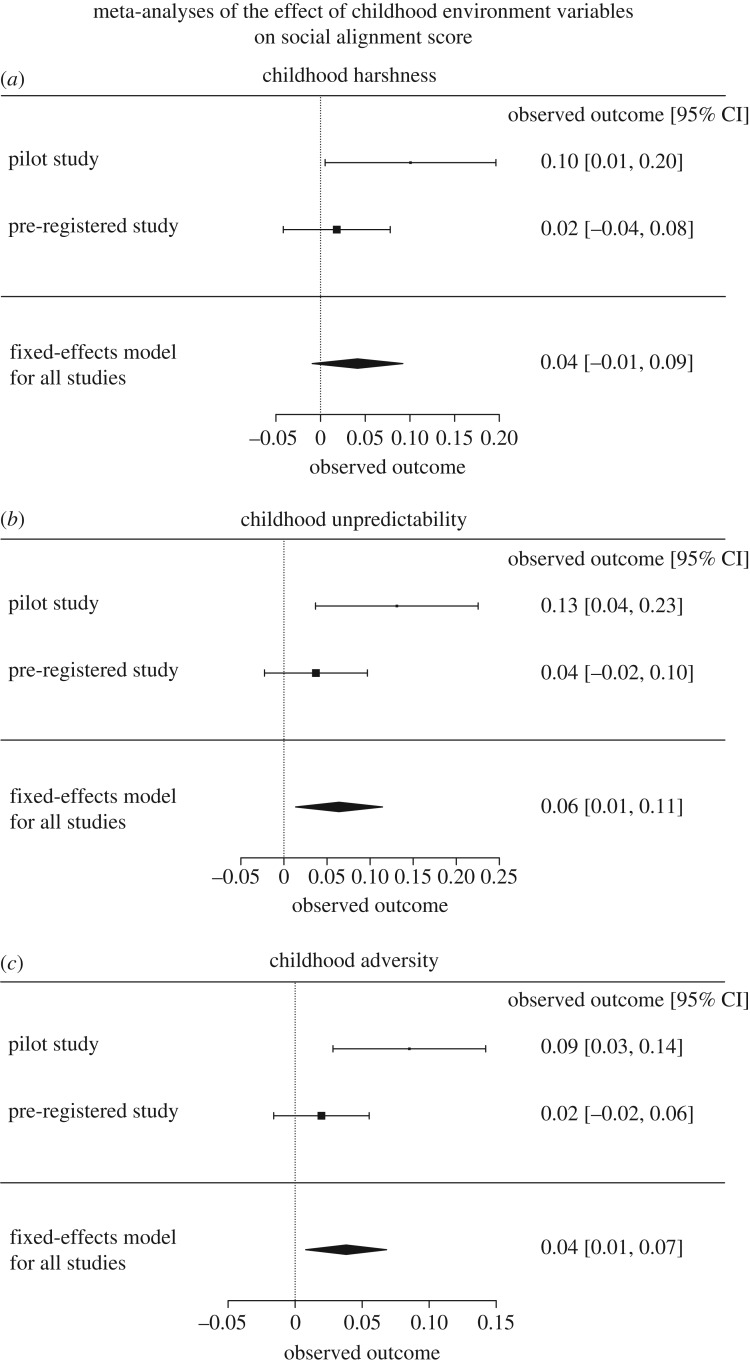
Meta-analyses of the effect of childhood environment variables on social alignment scores. Forest plots for the three meta-analyses: (*a*) childhood harshness, (*b*) childhood unpredictability, (*c*) childhood adversity. The results of the individual studies are shown on the right part of the plots (observed outcome [95% CI]). Below each plot, a summary polygon shows the results from a fixed-effects model when analysing the pilot and the pre-registered studies.

We started by meta-analysing the main effect of childhood harshness on social alignment scores across the pilot study and the pre-registered study (figure 5*a*). The model revealed that social alignment scores tended to be related to childhood harshness (*β* = 0.04 ± 0.026, *z* = 1.60, *p* = 0.11, *CI lower* = −0.009, *CI upper* = 0.092). Childhood unpredictability was significantly associated with high social alignment scores (*β* = 0.06 ± 0.028, *z* = 2.48, *p* = 0.01, *CI lower* = 0.014, *CI upper* = 0.114) (figure 5*b*). The same result was found for the main effect of childhood adversity on social alignment scores (*β* = 0.04 ± 0.015, *z* = 2.47, *p* = 0.01, *CI lower* = 0.008, *CI upper* = 0.07) (figure 5*c*). Tests for heterogeneity showed that the true effects in the two studies were not significantly different (*Childhood Unpredictability*: *Q* = 2.72, *df* = 1, *p* > 0.05; *Childhood Adversity*: *Q* = 3.66, *df* = 1, *p* > 0.05). The respective contribution of the pre-registered study in the estimated averaged effects of childhood unpredictability and childhood adversity was 71% and 72%, respectively. Note finally that the averaged effects of childhood unpredictability and childhood adversity were robust to the inclusion of the random factor ‘study’ in the model (using maximum-likelihood estimator) (*Childhood Unpredictability*: *β* = 0.07 ± 0.03, *z* = 2.32, *p* = 0.02, *CI lower* = 0.01, *CI upper* = 0.12; *Childhood Adversity*: *β* = 0.04 ± 0.02, *z* = 2.03, *p* = 0.04, *CI lower* = 0.002, *CI upper* = 0.09).

Very similar results were produced by the meta-analysis of the social influence parameter *δ*. Indeed, a fixed-effects model showed that the average effects of childhood unpredictability (*β* = 0.03 ± 0.01, *z* = 2.37, *p* = 0.018, *CI lower* = 0.004, *CI upper* = 0.046) and childhood adversity (*β* = 0.03 ± 0.01, *z* = 2.75, *p* = 0.006, *CI lower* = 0.009, *CI upper* = 0.054) on social alignment scores were significant. These effects were also robust to the inclusion of the random factor ‘study’ in the model (using maximum-likelihood estimator) (*Childhood Unpredictability*: *β* = 0.03 ± 0.01, *z* = 2.36, *p* = 0.02, *CI lower* = 0.004, *CI upper* = 0.046; *Childhood Adversity*: *β* = 0.03 ± 0.01, *z* = 2.75, *p* = 0.006, *CI lower* = 0.009, *CI upper* = 0.054). Tests for heterogeneity also showed that the true effects in the two studies were not significantly different (*Childhood Unpredictability*: *Q* = 2.12, *df* = 1, *p* > 0.05; *Childhood Adversity*: *Q* = 0.33, *df* = 1, *p* > 0.05). The respective contribution of the pre-registered study in the estimated averaged effects of childhood unpredictability was 71%. However, it was only 7% for childhood adversity.

Note finally that even though the results of the pilot study and pre-registered study taken independently showed that childhood unpredictability did not significantly impact the noise parameter *σ*, such an effect was detected once the data of the two studies were pooled together (*β* = 0.04 ± 0.02, *z* = 2.30, *p* = 0.02, *CI lower* = 0.006, *CI upper* = 0.076).

#### 3.2.2. Slope outliers detection

The results of the meta-analysis described above suggest that the effects of interest might be undetected in the pre-registered study because of an important noise component in the data, which partly comes from the skewed distributions of unpredictability and harshness scores (see electronic supplementary material, figures S2 and S10 for a representation of the distributions of scores in environmental variables, as well as electronic supplementary material, figures S4 and S13 for their correlations with social alignment scores). Indeed, in both the pilot study and the pre-registered study, about a third of participants reported having experienced no unpredictability at all during their childhood. In the remaining score ranks, the number of observations was around 5 on average for the pilot study, and 10 for the pre-registered study. Because noise is unequally distributed between the different ranks of the childhood unpredictability axis (see electronic supplementary material, figures S2 and S10, in which residuals are plotted against environment variables' score ranks), there is a greater chance that the regression slopes will be biased by outliers. Note that a similar problem applies to the distribution of harshness scores. Unlike the distribution of unpredictability scores, however, the distribution of harshness scores is negatively skewed and/or bimodal. In sum, even if our hypothesis had been valid, the presence of slope outliers might have made it particularly difficult to test.

To overcome this problem, we first detected in both the pilot and the pre-registered datasets those of the participants who were outliers in the type 1 models regressing social alignment scores on harshness and unpredictability scores (pilot study: ‘childhood’ scores; pre-registered study: ‘childhood’ and ‘current’ scores). For each regression (pilot study: *n* = 2; pre-registered study: *n* = 4), data points with a Cook's distance greater than a standard cut-off of 4/*N*_participants_ were identified [57] (see electronic supplementary material, figure S15). Note that the removed data points can be located at different coordinates of the data space regardless of their positive or negative impact on the regression slopes. In total, 14 participants of the pre-registered dataset (which represents 5% of the original sample) were identified as slope outliers in at least one regression and were thus removed from the pre-registered dataset. By comparison, only two participants of the pilot study were identified as slope outliers (which represents 1.5% of the original sample), hence confirming that results of the pre-registered study are more corrupted by noise than results of the pilot study. We then ran the very same analyses on the pre-registered dataset excluding the 14 slope outliers. If the effects of environmental variables on social alignment scores are true positives, as the meta-analysis suggests, this procedure should offer us a better chance to detect them.

#### 3.2.3. Re-analysing the pre-registered dataset without slope outliers

**Figure 6.**
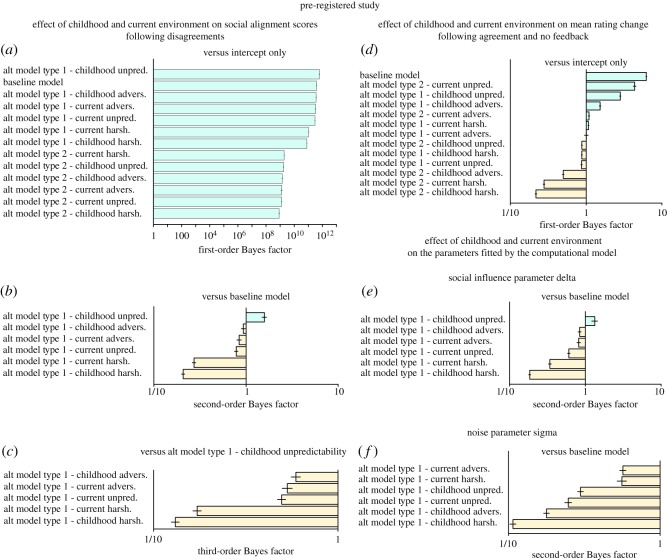
Unregistered analyses. Bayesian analyses of models with and without environmental variables as predictors of social alignment score in disagreement trials (*a*–*c*), of mean rating change in agreement trials (*d*) and of the parameters delta (*e*) and sigma (*f*) fitted by our computational model. The baseline model does not include any environmental variable; alternative models include each environmental variable as a main effect (type 1) or as a term interacting with disagreement valence and disagreement strength (type 2) (*a*–*c*,*e*,*f*) or control conditions (*d*). First-order Bayes factors compare the baseline model and alternative type 1 and 2 models with a null model including the intercept only. Second-order Bayes factors compare alternative type 1 and 2 models with the baseline model. Third-order Bayes factors compare the childhood and current adversity models, the childhood and current harshness models, and the current unpredictability model with the childhood unpredictability model. A Bayes factor less than 1 indicates evidence in favour of the reference model.

After removing slope outliers, we found that models including environmental variables as independent fixed-effects predicted the observed data better than alternative type 2 models (first-order Bayes factor, figure 6*a*). The second-order Bayes factor further revealed that the model including childhood unpredictability as a main effect had a better fit than the baseline model (figure 6*b*). Childhood unpredictability was positively associated with social alignment scores in disagreement trials (*β* = 0.08 ± 0.03, *t*(246) = 2.60, *p* = 0.01) (see electronic supplementary material, figure S16), though the evidence was weak (*Childhood unpredictability* versus *Baseline*: *BF*_10_s = 1.58 ± 5.36%). In addition, the type 1 model that included childhood unpredictability had a greater explanatory power than type 1 models that included current harshness (*BF*_10_s = 5.81 ± 5.15%), current unpredictability (*BF*_10_s = 2.02 ± 5.01%) or current adversity (*BF*_10_s = 1.88 ± 6.17%) (figure 6*c*). Finally, childhood environmental variables had no effect on control trials (agreement and no feedback trials): models including environmental variables did not outperform the baseline model (all *BF*_10_s < 0.44) figure 6*d*). Note that the type 1 model including childhood unpredictability as the main predictors performed better than similar models that included age (*BF*_10_s = 6.04 ± 6.06%) or education as predictors (*BF*_10_s = 12.23 ± 5.01%). A notable exception arose with the type 1 model including gender as a two-level group factor. This model outperformed the childhood unpredictability type 1 model, even though in a weak extent (*BF*_10_s = 1.58 ± 6.03%). Model parameters revealed that females had lower social alignment scores than males (*β* = −0.16 ± 0.06, *t*_246_ = −2.81, *p* = 0.005).

Identical results were revealed when the social influence parameter *δ* was taken as the dependent variable (*Childhood unpredictability* versus *Baseline*: *BF*_10_s = 1.33 ± 8.7%) (figure 6*e*), with a positive association between the parameter and childhood unpredictability (*β* = 0.03 ± 0.01, *t*_246_ = 2.54, *p* = 0.01). Again, this model provided more evidence than models which included current harshness (*BF*_10_s = 3.91 ± 9.12%), current unpredictability (*BF*_10_s = 2.20 ± 8.93%) or current adversity as the main predictor (*BF*_10_s = 1.63 ± 9.58%). Finally, none of the environmental variables included in the type 1 and type 2 models affected the noise parameter *σ* (all *BF*_10_s < 0.56, all *β*s < 0.06, all *t*s < 1.80, all *p*s > 0.07) (figure 6*f*).

## 4. Discussion

This research aimed to investigate the impact of individuals' ecology on their propensity to follow other people's opinion when making social judgements about strangers. We expected early-life environmental conditions to alter participants' sensitivity to peers’ opinion. This hypothesis was notably fuelled by two main sets of existing findings. First, evolutionary models converge on the idea that social modes of information acquisition are favoured to the extent that individuals' ecology conveys risks that hinder individualistic modes of information gathering, i.e. a strategy that brings higher pay-offs in the long run. Second, recent findings in human behavioural ecology suggest that early environmental stress exposure drives the adaptive calibration of individuals’ psychology towards short-term rather than long-term benefits [22] in various domains such as health, reproduction, parenting, economic decision-making or cooperation [23]. We reasoned that because the reliance on social information is a strategy that conveys immediate pay-offs (individuals exploit solutions that have already been successfully tried out by conspecifics), it should be affected by variations in people's ecology. We therefore tested the impact of two fundamental dimensions of early-life environmental risks—harshness and unpredictability—(as well as their combination) on people's susceptibility to social influence within two independent samples recruited online.

We initially used an analytic plan based on linear mixed models whose predictive value was further compared using Bayes factors. Results obtained in the pilot dataset (*N* = 121) showed a positive main effect of childhood unpredictability on participants' susceptibility to social influence: the more unpredictable the participant's childhood environment was, the more they aligned their social judgements about unknown faces with the modal judgements provided by other people. Moreover, combining measures of childhood harshness and childhood unpredictability into a single index of early-life adversity led to an even stronger effect than the two dimensions taken in isolation. Finally, a canonical computational model of choice showed that these effects were more likely accounted for by an increased motivation to match other people's choices, rather than by a corruption of internal representations by noise.

The relationship between the environmental variables and social alignment scores went in the same direction in the pre-registered analyses run on the replication sample (*N* = 262), but did not reach statistical significance. A meta-analysis conducted on both datasets revealed that childhood unpredictability and childhood adversity were significantly related to participants' susceptibility to follow other people's opinion, while the average effect of childhood harshness was close to significance. We therefore went beyond the pre-registered analyses and tried to identify the reasons why these effects were not directly detected in the replication dataset. The characteristics of the populations tested in both the pilot study and the pre-registered study presented important shortcomings that we had not anticipated. Both datasets were notably characterized by extremely skewed distributions of childhood unpredictability raw scores, which led to an unequal residual variance in social alignment scores across the different score ranks of the childhood unpredictability axis. The fact that noise is unequally distributed between the different ranks of the childhood unpredictability axis (see electronic supplementary material, figure S2 and S10, in which residuals are plotted against environment variables' score ranks) increases the chance that the regression slopes are biased by outlier data points. Similar problems were also observed with harshness scores. In sum, even if our main hypotheses had proven valid, the properties of the pre-registered dataset itself may have prevented us from testing them properly.

To check this possibility, we first looked at both datasets to identify outlier participants on the type 1 models regressing social alignment scores on environmental harshness and unpredictability. We found less than 1.5% outlier participants in the pilot study, but more than 5% outlier participants in the pre-registered study, which confirmed that the pre-registered dataset was more corrupted by noise. To counteract these limitations, we removed slope outliers following a standardized procedure of noise minimization [57] and ran the same analyses on this new set of participants (*N*_final_ = 262–14 = 248). These unregistered analyses revealed an effect of childhood unpredictability on participants’ performance, hence confirming the result obtained in the pilot dataset. Second, results showed that the effect of childhood unpredictability on social alignment scores was greater than the effect of unpredictability experienced by the participants at the time of testing.

In the next sections, we review our initial hypotheses in the light of the results obtained from the pre-registered and the unregistered analyses.

### 4.1. Main hypothesis #1: childhood unpredictability, harshness and/or adversity are positively associated with social alignment scores in disagreement trials, but not in control trials

Results of the pre-registered and the unregistered analyses detailed in this article converge on the fact that, overall, childhood environmental variables are not associated with mean rating change in control trials. On the other hand, information provided by the meta-analysis and by the analyses excluding slope outliers in the replication sample showed that childhood unpredictability and childhood adversity were positively associated with social alignment. Our main hypothesis #1 is therefore partially verified.

### 4.2. Main hypothesis #2: the effect of childhood environment on social alignment scores obtained in disagreement trials is caused by an increased valuation of social feedbacks, rather than by a corruption of participants' internal representations by noise

First, results of the analyses of the two datasets showed that childhood environment variables were unrelated to the noise parameter fitted by our computational model of choice. It is, however, important to note that an effect of childhood unpredictability on the noise parameter was detected once the data of the two studies were pooled together by the meta-analysis. Second, a positive relationship between the social influence parameter and childhood unpredictability scores/childhood adversity scores was found in the pilot study. These effects were also detected by the meta-analysis. We therefore conclude that our main hypothesis #2 is partially verified: an increase in childhood unpredictability and in childhood adversity, but not childhood harshness, lead to an increased valuation of conflicting social feedbacks from peers. However, we cannot entirely rule out the possibility that experiencing higher levels of unpredictability or adversity also contributes—albeit to a weak extent—to make internal representations noisier overall.

### 4.3. Exploratory hypothesis #1: childhood unpredictability has a greater impact on susceptibility to social influence than childhood harshness

In the pilot study, analyses showed that childhood unpredictability indeed had a greater impact than childhood harshness on social alignment score. The pre-registered analyses of the replication dataset failed to reveal such a hierarchy. However, the meta-analysis showed that the effect of childhood unpredictability averaged across both studies was significant, while the average effect of childhood harshness was not. This was further confirmed by the analyses of the replication dataset after excluding slope outliers, which revealed a significant effect of childhood unpredictability on the one hand, and an absence of effect of childhood harshness on social alignment scores on the other hand. Altogether, these results suggest that the exploratory hypothesis #1 can be considered valid.

### 4.4. Exploratory hypothesis #2: childhood adversity—the combination of childhood harshness and unpredictability—has a greater impact on susceptibility to social influence than its two dimensions taken in isolation

In the pilot study, childhood adversity had a greater impact on social alignment scores than childhood harshness and unpredictability taken independently. However, the same analyses performed on the replication dataset did not show such an effect. Neither the meta-analysis nor the replication analyses excluding slope outliers found this effect. Therefore, our exploratory hypothesis #2 is not verified.

### 4.5. Exploratory hypothesis #3: the effect of childhood environment on susceptibility to social influence is stronger than the effect of current environment

The analyses of the replication dataset after excluding slope outliers suggest that our hypothesis #3 is partly verified. Indeed, results showed that the model including childhood unpredictability as main predictor had a better fit than equivalent models with current harshness, unpredictability and adversity.

After having reviewed our working hypotheses in light of the results obtained from the pre-registered and the unregistered analyses, we now devote the next sections to potential limitations and ambiguities raised by our experimental design and results.

### 4.6. Better measures of childhood and current environment and a better representativeness of the participants’ samples are two necessary conditions to test the replicability of our findings in future studies

The most important limitation of our work undoubtedly concerns the way we measured participants' past and current environment and the characteristics of the two samples we recruited. Even though we used standardized questionnaires to assess environmental harshness and unpredictability [46–50], our analytic strategy was deeply constrained by the fact that a great number of the participants reported having experienced no unpredictability at all during their childhood or in their current life. To avoid the problems we met in the current work, future studies should therefore bring a particular attention to quantifying environmental harshness and unpredictability using more accurate tools and, if possible, to select populations whose individuals are more widely distributed on the harshness and unpredictability axes.

### 4.7. Potential interactions of environmental variables with other moderators of social influence

In our experiments, participants only knew that the social information was provided by other members of the MTurk community. In other words, they were not informed about the number of people who provided the social feedbacks, nor about their personal characteristics. This design was chosen on purpose. First, a simpler design allowed us to limit the number of independent variables and to focus on the relationship between environment and susceptibility to social influence. This strategy was further motivated by the fact that, to the best of our knowledge, this study is the very first one to systematically test these relations. Second, it has been shown that providing detailed information about the identity of the people composing the reference group could bias participants’ responses as a function of the strength of their affiliation (or non-affiliation) towards the group [58]. Finally, beyond a certain group size (typically 5–10 individuals) the strength of the influence exerted by the group has been shown to be relatively stationary [59]. Nevertheless, it would be interesting to investigate whether the effect of environmental adversity on susceptibility to social influence interacts with some characteristics of the people producing social information, such as their gender, their reputation, the social categories they belong to, etc.

### 4.8. Epistemic trust versus social trust

One of our main predictions was that experiencing an unpredictable environment during childhood would lead individuals to give more weight to social information later in life. This is based on the well-accepted idea that social information is cheaper than individual exploration [17]. However, one can argue that the opposite prediction could be made because, in harsh and unpredictable environments, individuals tend to be less cooperative [60] and therefore should be trusted to a lesser extent [61]. However, the task we used is not a cooperative task, and it does not involve social trust. In the context of our task, individuals are not producing information for the benefit of the participants. Peers' behaviour is simply available, and the observer may choose to copy them or not, just as in most real-life situations. There is no cooperation between the participant and the individuals she is observing, and thus no conflict of interest. In other words, it is important to distinguish between a situation of epistemic trust (i.e. believing in someone's expertise) and a situation of social trust (i.e. believing that someone is not going to cheat). The benefits of epistemic trust and social trust are not the same in a harsh and/or unpredictable environment: in such environments, epistemic trust should be high because information gathering is expensive but social trust should be low because cooperation is limited.

### 4.9. Selectivity of the effect of the environment on the sensitivity to negative social information

Another potential criticism is that one could expect the environment to have a selective effect on social information conveying negative social judgements (the face is judged by other people as less approachable than what the participant thinks), but not on social information conveying positive social judgements (the face is judged by other people as more approachable than what the participant thinks). This prediction is based on the idea that in harsh or unpredictable environments, levels of interpersonal trust and cooperation are lower, so it is more risky to follow others' opinion when the content is positive. Error-management theory predicts that, in such environments, individuals should evolve decision biases that lead them to choose the least costly option in case of error [62–65]. In the present task, positive and negative social judgements do not carry the same risks: positive judgements, while increasing potential benefits from cooperative interactions, increase the risk of being harmed or cheated. By contrast, negative judgements, because they lead individuals to avoid the interaction, do not carry the same risk of exploitation. Consequently, participants who experienced higher levels of unpredictability should accept negative judgements more easily. This is not what we observed in our data. This is particularly noteworthy because the error-management decision bias described above is indeed present in the results of the pre-registered dataset: the valence of the disagreement with other people has a main effect on social alignment scores, with participants being more prone to change their ratings in line with the group when the group provided a more negative rating on first sight. In a recent work using a similar task to the one we used here, we found that the effect of disagreement valence on social alignment scores interacted with how much participants felt vulnerable to extrinsic morbidity risks [65]. These mixed findings might be an indication that a sensitivity to negative social information might be indeed present in the general population, but might be specifically calibrated by the individuals’ responsivity to morbidity threats—a trait which can be under genetic influence and therefore relatively independent of an individual's ecology [66,67]—instead of environmental harshness or unpredictability levels.

### 4.10. Generalizability of the effect of the environment on susceptibility to social influence to non-social decision-making domains

These observations raise the question of the generalizability of the effect of the environment on susceptibility to social influence. Does this effect apply to decisions in the social domain only? Our opinion is that early adverse environments might indeed lead to a higher susceptibility to social influence in a variety of decision-making domains (e.g. social, perceptual, economic, politics). The reason is that other people's decisions can have an epistemic value in other domains than the social one (e.g. perceptual, economic, etc.). Results of a few recent studies using ecological priming techniques are in line with this view [68,69]. These studies both demonstrated that priming participants with morbidity cues led to an increased reliance on social information in task contexts that required non-social decisions.

After having listed the main limitations of our results and their potential ambiguities, we wish to close the discussion by a description of the potential scope of the findings detailed in this paper.

### 4.11. Unpredictability of the environment, not harshness, impacts the participants' susceptibility to social influence

One of our main hypotheses was that childhood unpredictability should have a greater impact than childhood harshness on participants’ susceptibility to social influence. This is precisely what we observed. However, we also predicted that childhood harshness would affect social alignment scores, which it did not. This result is somewhat in contradiction with recent empirical data showing that priming participants with external morbidity cues—a fundamental component of environmental harshness—increased their propensity to match their decisions with those made by a group of reference [70,71]. Another recently published study conducted by some of us and using an experimental design similar to the one used in this work also found that participants' perceived vulnerability to morbidity risks was positively associated with susceptibility to social influence [72]. We further demonstrated that the strength of this association was mediated by an increased neurophysiological response (measured by electro-encephalography) to conflicting feedbacks from peers. In parallel, results of the meta-analysis and of the robust analyses showed that higher levels of childhood unpredictability were consistently associated with higher levels of social alignment score.

How can we explain the absence of effect of environmental harshness? A first possibility is that environmental harshness measured in this work is derived from the participants’ childhood and/or current socio-economic status (SES). Even though SES has been shown to vary with almost every type of environmental risks including morbidity risks, it is possible that this measure is too vague to capture the effect conveyed by more specific morbidity factors (extrinsic or intrinsic). An alternative view is that the differential effect of harshness and unpredictability found in this work provides empirical support to theoretical views assuming that harshness—at least when represented by the level of economic resources—imposes tractable morbidity and mortality threats that can be somewhat buffered by the maintenance of long-term behavioural strategies that shield individuals from predictable fitness costs [24]. By contrast, unpredictability increases the temporal variance of morbidity and mortality risks, making environmental cues inconsistent to predict the future, and therefore favours present-oriented behaviours in response to stochastic variations of the environment [24].

### 4.12. Is there a specific developmental time-window during which the brain calibrates sensitivity to social information?

An important point of discussion concerns the developmental timing during which a relatively persistent sensitivity to social information is shaped. Our initial hypothesis was that the development of a consistent reliance on social information should be based on environmental cues processed during the first years of life (here from 0 to 10 years of age). Our reasoning was based on a number of works demonstrating that the early-life period is a privileged window during which organisms make decisions about the best way to allocate energetic resources among different competing biological activities (e.g. growth versus reproduction) [70,71,73,74]. These works also showed that the timing and pattern of resource allocation are partly determined by the informational content of the environmental cues, i.e. whether the cues represent stable states of the world that the organisms are likely to encounter later in life. Our results are strictly coherent with this view. Our findings suggest that childhood is a critical period during which our susceptibility to social influence is partly calibrated in a way that is adapted to environmental challenges met during development.

### 4.13. Ecological conditions contribute to shape individuals' reliance on social information

Overall, our work extends on a number of recent studies showing that individuals who felt particularly vulnerable to extrinsic morbidity risks were also more likely to conform their opinion to that of the majority [68,69,72]. Nevertheless, our contribution goes beyond these findings on several aspects. First, our work reveals that the ecology experienced by an individual throughout her lifespan is a putative cause of this association. Second, it suggests that the differential susceptibility to social influence can be better understood as a plastic response of the phenotype to external contingencies. Third, it shows that the preference for a social mode of information acquisition can emerge not exclusively as a response to pathogen threats but as a response to other dimensions of environmental risks, such as environmental unpredictability. The theoretical consequences of our work are not trivial. Notably, it provides an interesting alternative to the idea that the functionality of susceptibility to social influence is restricted to the protection of individuals from risks of pathogenic contamination [66,69,75]. Instead, our findings are more in line with modelling works of the evolution of social learning strategies, which state that social mode of information gathering should be favoured in ecological settings in which personal exploration conveys important fitness costs to the individuals [17,76]. These costs can undoubtedly be imposed by morbidity risks, but the incidence of other ecological (including psycho-social) variables should not be neglected as long as they contribute to the overall predictability of the environment.

To conclude, this work is, to the best of our knowledge, the first to provide experimental evidence for an association between individuals’ past and current ecology and their susceptibility to social influence. We hope that this paper will motivate future studies to replicate our findings and to go further in the topics. We believe that their outcomes might have important implications for our understanding of the evolution of human cultures [77]. In particular, it might explain differences in conformism and individualism between poor and affluent societies [78] as well as the modern rise of intellectual freedom and non-conformism in industrial and post-industrial societies [79,80].

## Supplementary Material

Supplementary Information

## Supplementary Material

Data_MatlabScripts_RScripts_PilotStudy_ReplicationStudy

## Data accessibility

The raw datasets of both the pilot study and the pre-registered replication, as well as the Matlab and R scripts used to pre-process and analyse them, are available from the Open Science Framework at https://osf.io/6etf3/ and from the Dryad Digital Repository at https://datadryad.org/resource/doi:10.5061/dryad.k48mr0c [81].
