# Supplementary material for: The ecological roots of human susceptibility to social influence: a pre-registered study investigating the impact of early-life adversity
Source: R Soc Open Sci. 2019 Jan 9;6(1):180454. doi: 10.1098/rsos.180454 (PMC6366227; doi:10.1098/rsos.180454)
Supplement: Supplementary Information [file rsos180454supp1.pdf]

– Registered report –

The ecological roots of human susceptibility to social influence: a pre-registered study investigating the impact of early life adversity

Pierre O. Jacquet[1,2], Lou Safra[1], Valentin Wyart[1], Nicolas Baumard[2], Coralie Chevallier[1]

[1] Laboratoire de neurosciences cognitives, Département d'études cognitives, École normale supérieure, INSERM, PSL Research University, 75005 Paris, France.

[2] Institut Jean Nicod, Département d'études cognitives, ENS, EHESS, CNRS, PSL Research University, 75005 Paris, France.

Supplementary Information

1. Pilot study

1.1. Supplementary results

1.1.1. Distributions of mean rating change in each type of disagreement trials

The Supplementary Figure S1 shows the distributions of mean rating change in each type of disagreement trials (moderate/negative, strong/negative, moderate/positive, strong/positive).

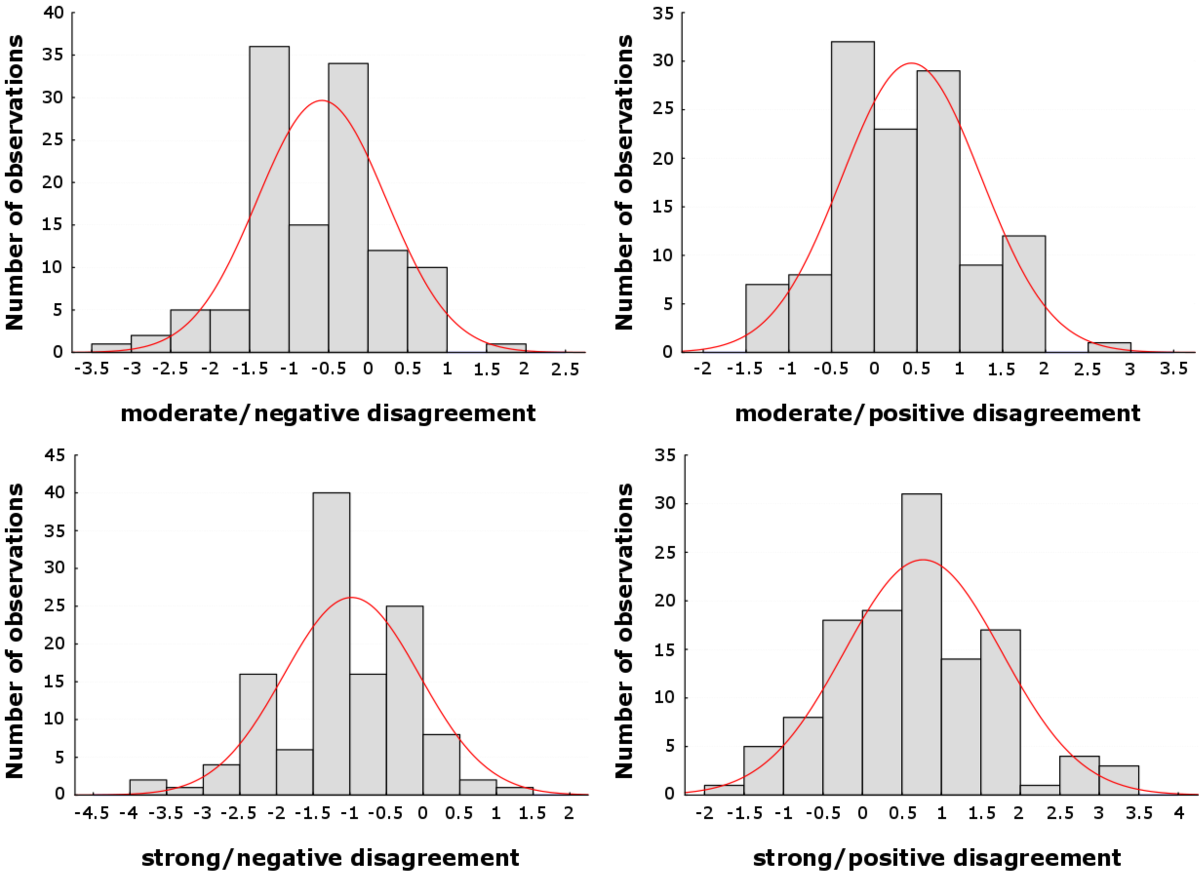

Supplementary Figure S1. Distributions of mean rating change in each type of disagreement trials

1.1.2. Distributions of scores in environment variables and their relation with residuals

The Supplementary Figure S2 shows the distributions of childhood harshness and unpredictability scores as well as their relation with residuals.

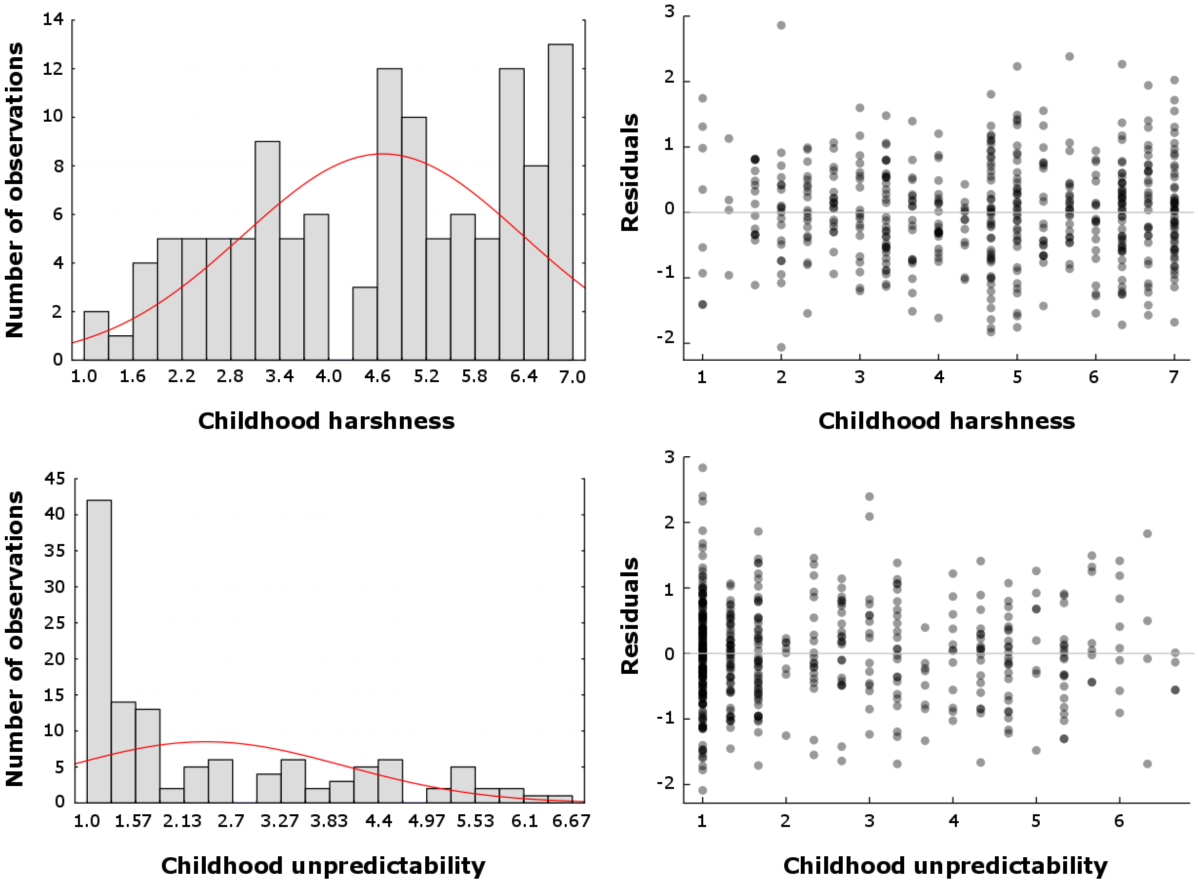

Supplementary Figure S2. Distributions of childhood harshness and childhood unpredictability scores, and their relation with residuals.

1.1.3. Correlation of mean rating change and environmental variables for each type of disagreement trials

The Supplementary Figure S3 shows the correlations of participants' performance in terms of mean rating change and childhood harshness, unpredictability and adversity, respectively. The correlation matrix with *r* coefficients is also inserted below (Supplementary Table S1).

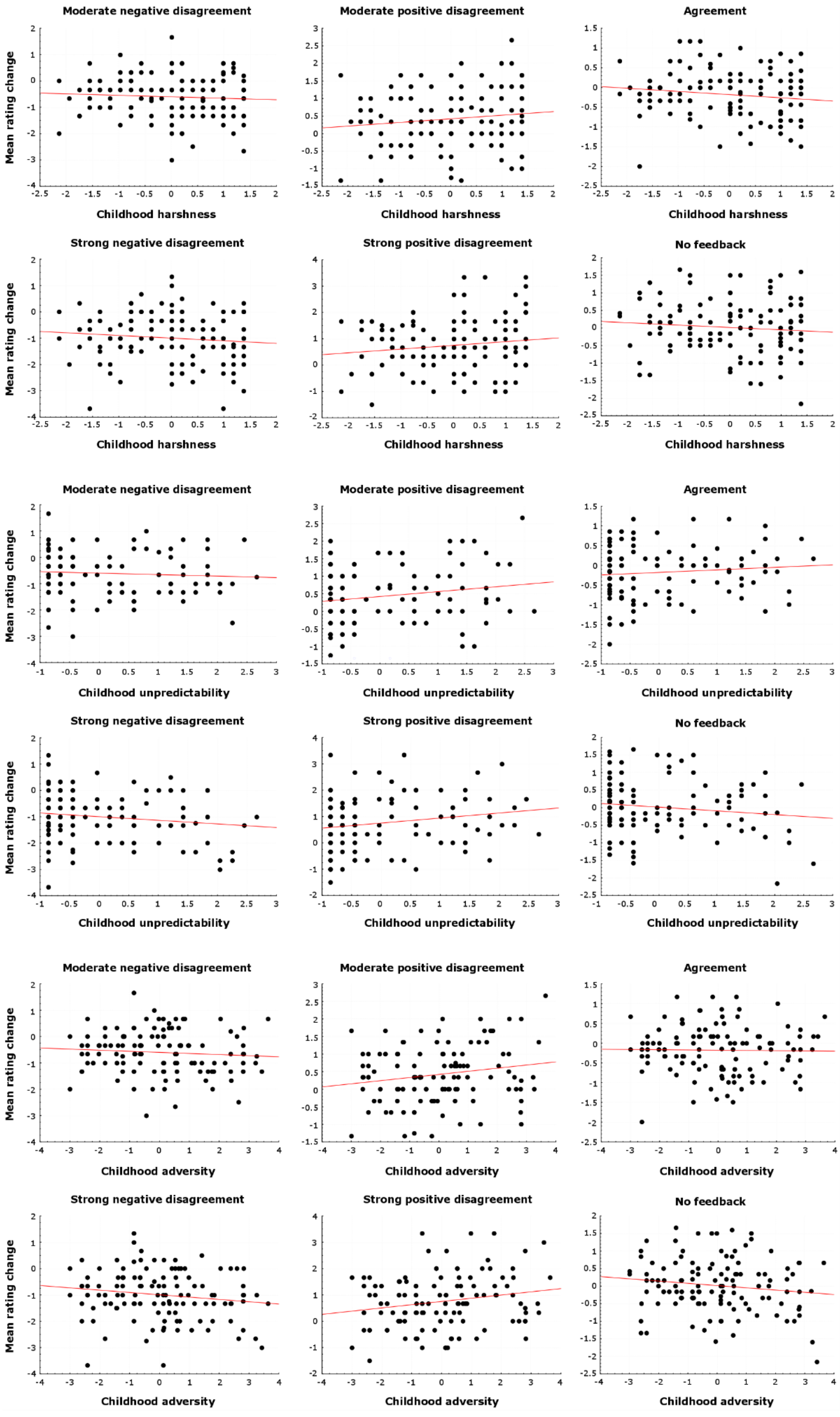

Supplementary Figure S3. Correlation of mean rating change obtained in each type of disagreement trials and environment variable (childhood).

1.1.4. Correlation of social alignment scores and environmental variables for disagreement trials

The Supplementary Figure S4 below shows the correlations of participants' performance in terms of social alignment score and childhood harshness, unpredictability and adversity, respectively. The correlation matrix with *r* coefficients is also inserted below (Supplementary Table S1).

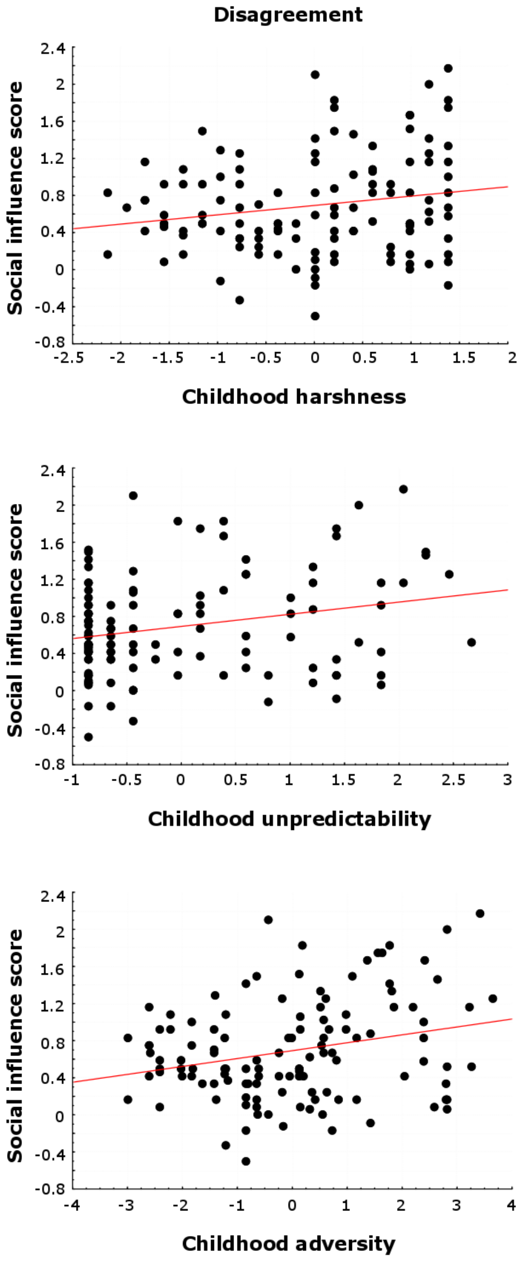

Supplementary Figure S4. Correlation of social alignment scores obtained in disagreement trials and environment variables (childhood).

				Childhood harshness	Childhood unpredictability	Childhood adversity
Mean rating change	Disagreement trials	negative	moderate	-0,07	-0,07	-0,08
			strong	-0,11	-0,15	-0,16
		positive	moderate	0,13	0,17	0,18
			strong	0,14	0,19	0,2
	Agreement trials			-0,13	0,1	-0,02
	No feedback trials			-0,09	-0,14	-0,14
Social alignment score	Disagreement trials			0,19	0,24	0,26

Supplementary Table S1. Correlation matrix (*r* coefficients).

1.1.5. Testing the adequacy of the computational model of choice

All the effects revealed by the analyses of the participants' social alignment scores were also obtained with the model's predictions (figure 2.b of the main manuscript). Using *t*-tests for single mean and *t*-tests for dependent samples, we first showed that the model accurately predicts an increase in social alignment scores in the positive disagreement trials of moderate ($M = 0.42 \pm 0.81$, $t(121) = 5.72$, $p < .001$) and bigger strength ($M = 0.75 \pm 1.00$, $t(121) = 8.29$, $p < .001$), and in the negative disagreement trials of moderate ($M = 0.60 \pm 0.81$, $t(121) = 8.12$, $p < .001$) and bigger strength ($M = 1.00 \pm 0.92$, $t(121) = 11.87$, $p < .001$) (figure 2.b of the main manuscript).

Moreover, a linear mixed model ran on model-predicted social alignment scores, and including disagreement valence and disagreement strength as fixed factors, and participants' ID as a random factor, showed the same pattern of results than an equivalent model ran on real scores. Indeed, the computational model predicted a main effect of the disagreement strength factor, with greater scores in the moderate than strong disagreement trials ($\beta = 0.28$

± 0.02, $t(480) = 12.71$, $p < .001$). Neither the valence of the disagreement nor its interaction with the strength had an effect on social alignment scores.

Finally, the model-predicted scores explained a significant component of scores calculated from behavioural data (moderate positive disagreement: adjusted $R^2 = .30$, $df = 119$, $F = 52.74$, p < .001; strong positive disagreement: adjusted $R^2 = .46$, $df = 119$, $F = 102.70$, p < .001; moderate negative disagreement: adjusted $R^2 = .27$, $df = 119$, $F = 44.54$, p < .001; strong negative disagreement: adjusted $R^2 = .42$, $df = 119$, $F = 87.89$, p < .001; Supplementary Figure S5).

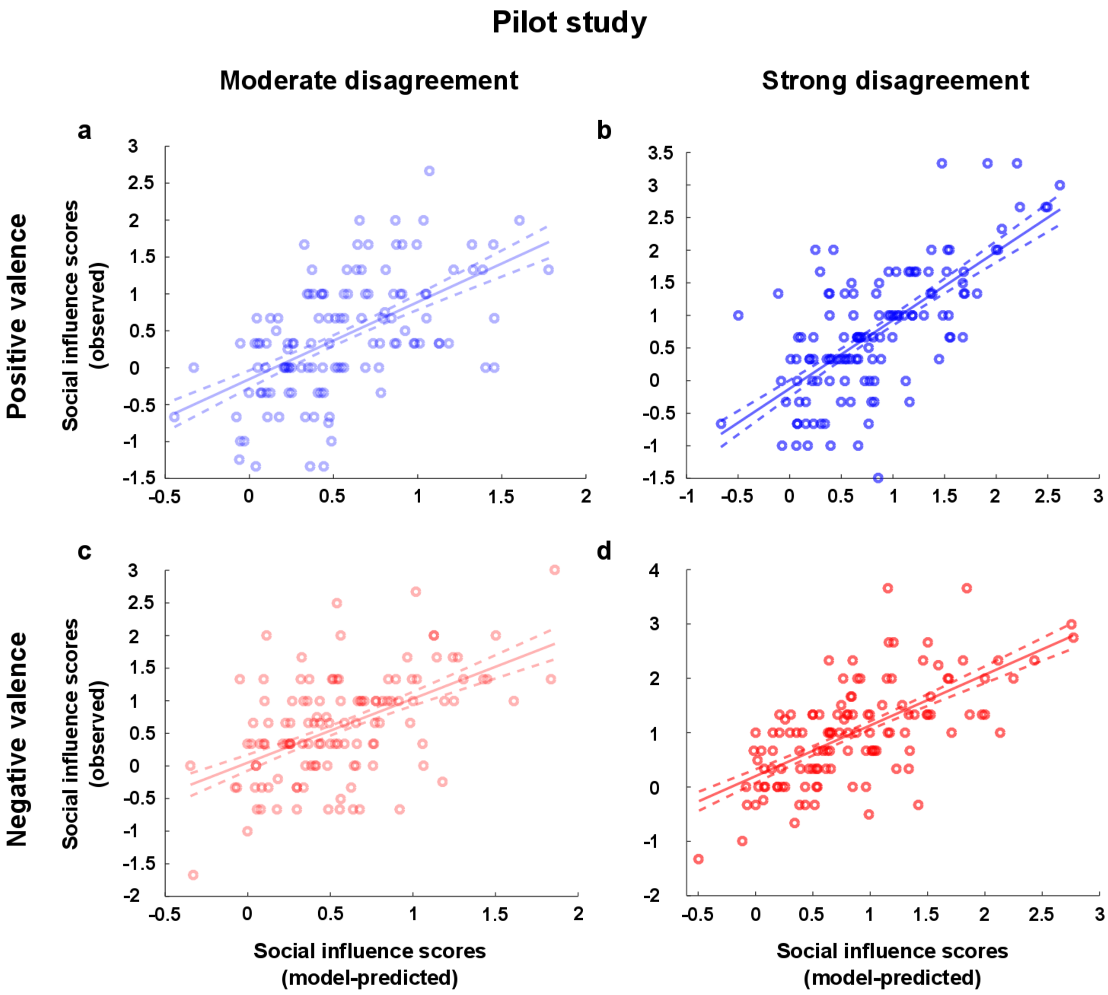

Supplementary Figure S5. Pilot study. Observed adjustment scores regressed on model-predicted social alignment scores. Positive disagreement of **a.** moderate and **b.** strong magnitude. Negative disagreement of **c.** moderate and **d.** strong magnitude.

2. Pre-registered study

2.1. Materials and Methods

2.1.1. Stimuli and Procedure

The only difference to the experimental procedure is a change in the distracting task separating the test and the post-test phase. Even though the images used in the pilot studies were judged as neutral (8-point scale: $M = 2.99$, $SD = 1.22$), their content might nonetheless interfere with the task. Therefore, instead of judging neutral images on the disgust dimension, participants engaged in a fully neutral task where they compared the surfaces of two squares, i.e., a grey square and a black square (Supplementary Figure S6). Eighteen trials were performed. The image featuring the two squares was presented for 3 seconds. The side on which the grey and black squares appear was counterbalanced across trials. A numerical scale then appeared on the screen until participants selected a value comprised between 1 and 8, representing how much bigger the surface of the black square is compared to the grey square (1 = same size, 8 = 8 times bigger). The square surface ratio was 1/2, 1/4, or 1/8. These ratios were chosen to minimize task difficulty.

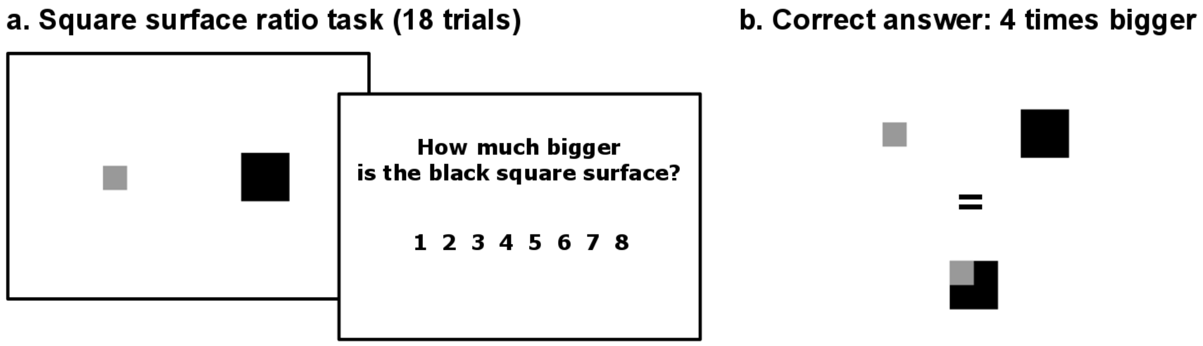

Supplementary Figure S6. Distracting task used in the pre-registered study: square surface ratio task. a. Participants were asked to estimate the ratio between the surface of a black square varying across trials, and the surface of a grey square held constant across trials. Eighteen trials were performed and the ratio between the two square surfaces varied from 1 to 8. Each image featuring the two squares was presented for 3 seconds. After each image, participants selected a value on an 8-point scale, representing how much bigger the surface of the black square is compared to the grey square (1 = same surface; 8 = 8 times bigger). **b.** In the example featured on the left panel of the figure, the black square surface is 4 times bigger than the grey square surface.

2.1.2. Assessing susceptibility to social influence

The analysis plan is identical to the one we used in the pilot study. Briefly, performance was first examined by looking at the mean difference between test and post-test approachability ratings. Mean rating change was computed in all trial types. Positive and negative mean rating changes indicate that participants either increase or decrease their approachability ratings in the post-test. A social alignment score was then computed following the same method as in the pilot study (see section 2.1.3. of the main manuscript). Positive and negative social alignment scores indicate that participants adjusted their ratings either *in line* with the group or *away* from the group respectively.

2.1.3. Assessing early life environment

Exposure to adverse environments was computed using the same unpredictability and harshness questionnaires [2-6] as in the pilot study. Individual item scores were averaged and z-scored following the procedure described in the pilot study (see section 2.1.4. of the main manuscript). As previously, the z-transformed unpredictability and harshness scores were summed in order to obtain a single score of childhood adversity [7] and the resulting adversity score was used to assess the synergistic effect of childhood environmental harshness and unpredictability on social influence.

2.1.4. Assessing current environment

In addition to the measures used in the pilot study, we also assessed participants' perception of their current level of environmental adversity. The three-item questionnaires mentioned above were adapted for this purpose [1-5]. Participants wereasked to assess the extent to which their current environment is unpredictable: "In the past few years: (a) things have often been chaotic in my life, (b) people in my neighborhood have often moved on a pretty random basis, and (c) I have had a hard time knowing what people in my family, in my neighborhood

or at work were going to say or do from day-to-day." Responses will be made on a 7-point scale, ranging from 1: strongly disagree, to 7: strongly agree. The same approach was used for current harshness. Participants were asked to assess the extent to which their current environment is scarce: "In the past few years: (a) My family and I have had enough money for things, (b) I have lived in a relatively wealthy neighborhood, (c) I have felt relatively wealthy compared to other people in my neighborhood." Responses will be made on a 7-point scale, ranging from 1: strongly disagree, to 7: strongly agree. Analyses of participants' answers to these questions were identical to the ones used to analyse childhood questionnaire scores.

2.1.5. Computational model description and fitting

The fitted computational model was fully identical to the one used in the pilot study (see section 2.2.5. of the main manuscript). Briefly, it consisted of two free parameters, fitted to each participant's responses: a social influence parameter δ and an internal noise magnitude parameter σ. The model computed maximum-likelihood estimates of the two parameters δ and σ separately for each participant.

2.2. Analytic strategy

2.2.1. Participants recruitment and online procedure

As in the pilot study, participants were recruited on-line via the Mechanical Turk platform and the study was coded using Qualtrics. Individual datasets were stored on the Qualtrics platform. Each dataset was submitted to the same quality checks as in the pilot study to ensure that *i*) the participant declared being 18 years old or more, *ii*) entered the correct verification code, *iii*) did the task only once and *iv)* had enough valid data in each experimental condition.

2.2.2. Data cleaning

As in the pilot data, participants who do not fulfill the quality checks were excluded as well as participants who were outliers (using the 1.5 times interquartile range criterion) on social alignment score averaged across all types of disagreement, or on harshness and unpredictability scores. In the pilot study, 22 out of 143 participants were excluded following these exclusion criteria, hence representing a total loss of approximately 15%. Excluded participants were not replaced. Instead, we increased our sample size estimation in proportion to the percentage of loss obtained in the pilot study.

2.2.3. Data quality checks and positive controls

A visual inspection of our dependent measures (social alignment scores obtained in each sub-dimension of the group disagreement) was carried out to check whether their respective distribution approximately matches the theoretical normal distribution. Skewness and kurtosis parameters are also reported. Visual inspection and descriptive statistics of outcome measures' distribution were crossed with Shapiro-Wilk's W tests to specify their normality. Finally, a Levene's test of the homogeneity of variances was conducted to check the assumptions of the general linear model. In addition, the validity of childhood/current unpredictability and childhood/current harshness constructs was assessed using inter-item reliability analyses. We expected unpredictability and harshness questionnaires to present a satisfactory internal consistency, i.e. *Cronbach's alpha* >= .70.

Finally, participants' mean rating change (the mean difference between post-test approachability ratings and test approachability ratings) was compared across trial types to ensure that group disagreement has a selective impact on participants' ratings adjustment.

2.2.4. Hypotheses testing and expected results

As in the pilot study, all analyses were performed using Matlab and R. We ran a set of linear mixed models that we then compared using Bayes factors (see section 2.2. of the main manuscript). All models used a maximum likelihood fitting method and had random intercepts. As a preliminary step for model comparison, a linear mixed model was run with social alignment score as the dependent measure and testing the main effects of disagreement valence and disagreement strength as well as their interaction effect. Disagreement valence (negative vs. positive) and disagreement strength (moderate vs high) were entered as within-subject fixed-effect factors, and participants' ID as a random factor. This model served as a baseline for model comparison analyses (see below).

Our hypotheses were tested using the following analysis plan:

- **Main hypothesis #1.** Childhood unpredictability, harshness and/or adversity are positively associated with social alignment scores in disagreement trials, but not in control trials.

  Statistical analyses. We applied exactly the same statistical analyses as that used in the pilot study. The contribution of childhood adversity and of its two dimensions – harshness and unpredictability – on participant's social alignment scores in disagreement trials was investigated by comparing the baseline model with alternative models including the childhood environment variable of interest as a predictor either as a main effect (alternative type 1 model) or as a term interacting with disagreement valence and disagreement strength (alternative type 2 model). A first-order Bayes factor was calculated for each model, providing an indication of the predictive power of these models relative to a null model including the intercept only. A second-order Bayes factor further compared the predictive power of alternative models with the baseline model,

taken as the reference model. A greater Bayes factor for one of the alternative models relative to the baseline model would indicate evidence for an effect of childhood environment on social alignment scores.

A similar strategy was employed to investigate the effect of childhood environment on the mean rating change collected in the control conditions (agreement and no feedback). A baseline linear mixed-effects model taking mean rating change as the dependent variable, the type of control condition as the two-level fixed effect factor, and the participants' ID as a random factor, was first generated. We then compared this model with alternative type 1 and 2 models using Bayes factors.

Expected results. We expected alternative models taking social alignment scores as the dependent variable to yield greater Bayes factors than the baseline model. Given the results obtained in the pilot data, we also expected that, among the two alternative models, greater evidence would be found for the type 1 model including childhood environment variables as main effects (i.e., independently of disagreement valence and strength). The sign of the correlation between childhood environment variables and social alignment scores was further specified by the parameters and coefficients estimated by the linear mixed-effects models. This pattern of results should not be observed in the control conditions. We indeed predicted the baseline model to better fit the mean rating change than alternative models.

- **Main hypothesis #2:** The effect of childhood environment on social alignment scores obtained in disagreement trials is caused by an increased valuation of social feedbacks, rather than by a corruption of participants' internal representations by noise.

Statistical analyses. The statistical analyses used to test the main hypothesis #1 was also used to test the main hypothesis #2. Linear mixed models were however applied

separately on the social influence parameter δ and the noise parameter σ fitted by our computational model.

Expected results. Overall, we expected alternative models (with childhood environment variables) taking the social influence parameter δ as the dependent variable to yield greater Bayes factors than the baseline model (without childhood environment variables). Conversely, an opposite pattern of results should be observed for the comparison of models taking the noise parameter σ as the dependent variable, with the baseline model yielding a greater Bayes factor than alternative models.

- **Exploratory hypothesis #1.** Childhood unpredictability has a greater impact on susceptibility to social influence than childhood harshness.

  Statistical analyses. As in the pilot study, we tested this hypothesis by calculating a third-order Bayes factor comparing the best fitting alternative model found for childhood unpredictability with the best fitting alternative model found for childhood harshness, with social alignment scores obtained in disagreement trials taken as the dependent variable.

  Expected results. We expected a greater Bayes factor for models including childhood unpredictability.

- **Exploratory hypothesis #2:** Childhood adversity – the combination of childhood harshness and unpredictability – has a greater impact on susceptibility to social influence than its two dimensions taken in isolation.

  Statistical analyses. A third-order Bayes factor was calculated to compare the best fitting alternative model found for childhood adversity (here taken as the reference model) with the best fitting alternative model found for each of its dimension, with social alignment scores obtained in disagreement trials taken as the dependent variable.

Expected results. We expected a greater Bayes factors for models including childhood adversity.

- **Exploratory hypothesis #3:** The effect of childhood environment on susceptibility to social influence is stronger than the effect of current environment.

  Statistical analyses. The analytic strategy employed to test the main hypothesis and the exploratory hypotheses #1 and #2 was also applied to test the exploratory hypothesis #3. Alternative type 1 and 2 models included current instead of childhood environment variables as predictors. Models including current and childhood environment variables were compared using Bayes factor.

  Expected results. We expected models including childhood environment variables to have greater Bayes factors than models including current environment variables.

2.3. Power analyses and sample size estimations

2.3.1. Frequentist tests

Our estimation of the sample size (performed in GPower-3 [8]) required for the pre-registered study was based on the regression slopes obtained from the linear mixed models used to test the main hypotheses #1 and #2 in the pilot study, i.e., the positive main effects of childhood harshness, unpredictability and adversity on social alignment scores and on the fitted social influence parameter δ.

- **Main hypothesis #1.** In the pilot study (N = 121), the reported effect size (given by the estimated regression coefficient β) of childhood harshness, unpredictability and adversity on social alignment scores were respectively $\beta = 0.10$, $\beta = 0.13$ and $\beta = 0.09$. Post-hoc power analyses indicated an achieved power of 0.69 for childhood harshness, 0.89 for childhood unpredictability, and 0.92 for childhood adversity. The sample size

required to achieve a 95% power at an α-level of 5% is estimated at N = 282 for the effect of childhood harshness, N = 160 for the effect of childhood unpredictability, and N = 139 for the effect of childhood adversity.

- **Main hypothesis #2**. The reported size of the effects of childhood harshness, unpredictability and adversity on the fitted social influence parameter δ were respectively β = 0.04, β = 0.05 and β = 0.03. Post-hoc power analyses indicated an achieved power of 0.54 for childhood harshness, 0.68 for childhood unpredictability, and 0.76 for childhood adversity. The sample size required to achieve a 95% power at an α-level of 5% is estimated at N = 426 for the effect of childhood harshness, N = 294 for the effect of childhood unpredictability, and N = 236 for the effect of childhood adversity.

2.3.2. Bootstrapping data simulations

In addition to the frequentist sample size estimation tests, we performed bootstrapping data simulations on the pilot data to estimate how second-order Bayes factors derived from the comparison of linear mixed models without and with childhood environment variables as predictors (Baseline model vs. Alternative type 1 model, see sections 2.2.3., 2.2.4. and 2.3.5. of the main manuscript) changes as a function of an increasing sample size. In sum, these simulations provide an indication of the size of the effects that childhood environment variables (the Bayes factor magnitude) have on outcome measures, and that we might expect for sample sizes larger than the one used in the pilot study.

We first simulated the statistics we extracted from the analyses used to test the Main hypothesis #1. More specifically, a first set of simulations was performed on the second-order Bayes factor obtained from the comparison of the Baseline model and alternative type 1 model, taking as the dependent variable the social alignment scores following disagreement trials. The second set of simulations was performed on the second-order Bayes factor obtained from

the comparison of the Baseline model and the alternative type 1 model, taking as the dependent variable the mean rating change following agreement and no feedback trials.

Second, we simulated the statistics we extracted from the analyses used to test the Main hypothesis #2. More specifically, we performed simulations on the second-order Bayes factor obtained from the comparison of the Baseline model and alternative type 1 model, taking as the dependent variable the social influence parameter δ fitted by our computational model (Supplementary figure 3.a). The second set of simulations was performed on the second-order Bayes factor obtained from the comparison of the Baseline model and alternative type 1 model, taking as the dependent variable the internal noise magnitude parameter σ fitted by our computational model.

Simulations were performed by repeatedly drawing random samples of participants with a fixed size. Starting with a sample of size 10, we drew 1000 random samples. For each of the 1000 samples, we calculated the Bayes factor from the model comparison of interest. We then repeated this procedure for larger samples, increasing sample size by 15 participants at each step until the maximum size of N = 120 was reached (121 participants were included in the analyses of the pilot data). We then averaged the Bayes factor obtained from the 1000 random simulations for each sample size, and fitted a logistic function on the data points with gradient descent using the 'interior-point' algorithm of the Matlab *fmincon* routine. The projections of the fitted curves give us an indication of effect sizes we might expect with larger sample sizes.

- **Main hypothesis #1.** Supplementary Figure 7.a shows that the size of the effect of childhood adversity on social alignment scores increased as long as the sample size increased (black line), and suggests that the largest effect size (BF_{10} = 6) could be reached with a sample of approximately N = 200 (red curve). Sample size also had an impact on the effect of childhood unpredictability on social alignment scores (dark grey

line). However, this impact was smaller than on the effect of childhood adversity, with the greatest Bayes factor (BF_{10} = 4) reached around N = 140 (blue curve). Finally, simulations showed an anecdotal impact of sample size on the effect of childhood harshness on social alignment scores (light grey line). The Bayes factor reached a plateau around N = 140 and never increased beyond 1, a value indicating no evidence in favor of any models (green curve). To summarize, effects size (the Bayes factor magnitude) we might have expected with sample sizes larger than the one used in the pilot study should have been roughly equivalent to those already reported in the main manuscript.

Importantly, our simulations provided evidence that sample size had no impact on the effect childhood environment variables on the mean rating change obtained in the control conditions, with Bayes factors uniformly under 1 (Supplementary Figure 7.b).

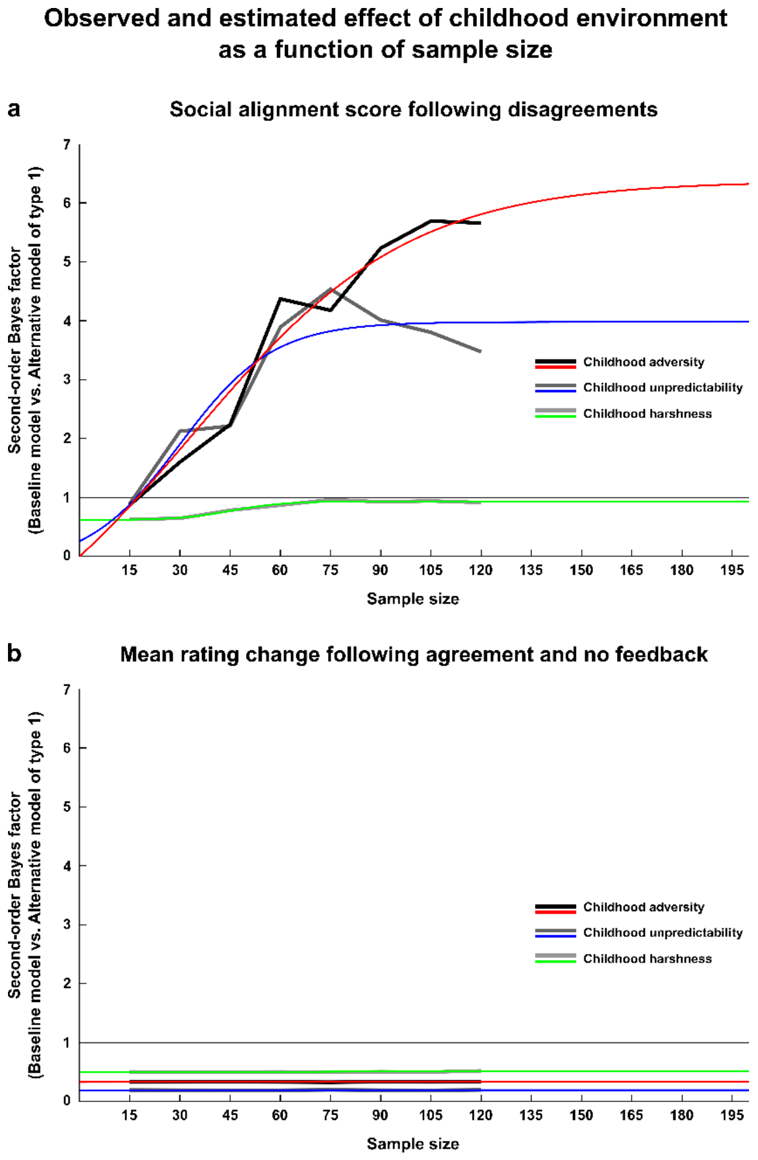

Supplementary Figure 7. Observed and estimated effect of childhood environment as a function of sample size. a. Effect of sample size (x axis) on Bayes factors (y axis) calculated from the comparison of models taking social alignment scores following disagreement trials as the dependent variable, with (Alternative type 1 model) and without childhood (Baseline model) environment variables as independent predictors (harshness: light grey curve; unpredictability: dark grey curve; adversity: black curve). **b.** Effect of sample size (x axis) on Bayes factors (y axis) calculated from the comparison of models taking the mean rating change following agreement and no feedback trials as the dependent variable, with (Alternative type 1 model) and without (Baseline model) childhood environment variables as independent predictors (harshness: light grey curve; unpredictability: dark grey curve; adversity: black curve). For both panels, each data point represents the Bayes factor averaged across 1000 random simulations performed on *n* participants. Sample size was increased by steps of 15, until a maximum of N = 120, which represents the full sample analyzed in the pilot study, minus one participant. The projection of logistic functions (harshness: green slope; unpredictability: blue slope; adversity: red slope) fitted on data points provide effect size estimates one might expect with larger sample sizes. A Bayes factor of 1 (black horizontal line) indicates that the Baseline model and the Alternative model have an equivalent predictive power. A Bayes factor inferior to 1 indicates greater evidence for the Baseline model. A Bayes factor superior to 1 indicates greater evidence for the Alternative model.

- **Main hypothesis #2.** Supplementary Figure 8.a shows that the size of the effect of childhood adversity on the social influence parameter δ fitted by our computational model increased as long as sample size increased (black line), and suggested that the largest effect size ($BF_{10} = 4$) could be reached with a sample of approximately N = 200 (red curve). Sample size also had an impact on the effect of childhood unpredictability on this parameter (dark grey line), though to a smaller extent. The greatest Bayes factor ($BF_{10} = 2$) is expected to be reached around N = 140 (blue curve). As for simulations performed on the statistics extracted from the analysis used to test the main hypothesis #1, simulations showed an anecdotal impact of sample size on the effect of childhood harshness on the social influence parameter δ (light grey line), with a Bayes factor of 1 stabilized around N = 130.

  A similar anecdotal impact of sample size was also observed for the effect of childhood adversity and unpredictability on the internal noise magnitude parameter σ (Supplementary Figure 8.b). Finally, this impact was null for the effect of childhood harshness.

To conclude, we expected to verify our main hypotheses #1 and #2 on a sample of participants larger than the one recruited for the pilot study, with effect sizes approximately equivalent to those reported in sections 2.3.3., 2.3.4., and 2.3.5. Our predictions were of course valid provided that the participants involved in the pilot study represented an unbiased sample of the general population.

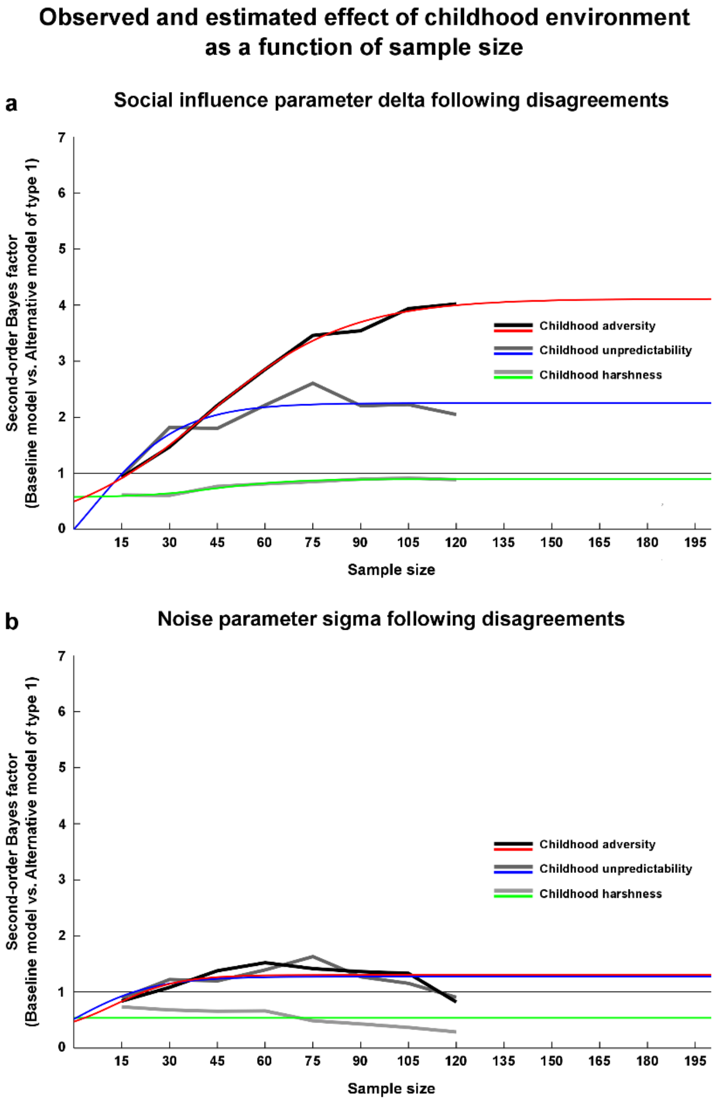

Supplementary Figure 8. Observed and estimated effect of childhood environment as a function of sample size. a. Effect of sample size (x axis) on Bayes factors (y axis) calculated from the comparison of models taking the social influence parameter δ fitted by the computational model following disagreement trials as the dependent variable, with (Alternative type 1 model) and without childhood (Baseline model) environment variables as independent predictors (harshness: light grey curve; unpredictability: dark grey curve; adversity: black curve). **b.** Effect of sample size (x axis) on Bayes factors (y axis) calculated from the comparison of models taking the internal noise magnitude parameter σ fitted by the computational model following disagreement trials as the dependent variable, with (Alternative type 1 model) and without childhood (Baseline model) environment variables as independent predictors (harshness: light grey curve; unpredictability: dark grey curve; adversity: black curve). For both panels, data points represent the Bayes factor averaged across 1000 random simulations performed on *n* participants. Sample size was increased by step of 15, until a maximum of N = 120, which represents the full sample analyzed in the pilot study, minus one participant. The projection of logistic functions (harshness: green slope; unpredictability: blue slope; adversity: red slope) fitted on data points provide effect size estimates one might expect with larger sample sizes. A Bayes factor of 1 (black horizontal line) indicates that the Baseline model and the Alternative model have an equivalent predictive power. A Bayes factor inferior to 1 indicates greater evidence for the Baseline model. A Bayes factor superior to 1 indicates greater evidence for the Alternative model.

Finally, bootstrapping simulations suggested that the evidence in favor of models including childhood harshness as predictor of social alignment scores or social influence parameter δ was likely to be null or anecdotal even with much larger sample sizes. Therefore, we discarded frequentist sample size estimations based on childhood harshness effects, and considered the remaining most conservative sample size estimation of N = 294 as the reference. Because in the pilot study we had a 15% loss of datasets, we increased the sample size required for the pre-registered study by n = 44. As a consequence, we planned to recruit 340 US adult participants on-line for the pre-registered study.

2.4. Complementary results

2.4.1. Participants included in the pre-registered protocol

We recruited 340 participants via Amazon Mechanical Turk (341 participants were finally able to complete the experiment). We found that 21 MTurkers had an IP addresses located outside US. Our initial sample therefore involved 320 US participants. Forty-five of them did not fulfill the quality checks, and 13 were outliers (using the 1.5 times interquartile range criterion) on social alignment scores averaged across all types of disagreement (no participant was an outlier on harshness or unpredictability scores). These 58 participants were excluded from the final analyses, representing a total loss of approximately 18% (a value close to the one we predicted, see section 2.3 of the Supplementary Information). We were thus left with a final sample of 262 individuals (154 females, 108 males; mean age = 38 ± 12).

2.4.2. Data quality check and data cleaning

Results of Shapiro-Wilk's W tests performed on mean rating change in disagreement trials rejected the null hypothesis that the data come from normal distributions (all Ws > .97, all ps < .001) (see Supplementary Figure S9 for frequency plots). Similarly, Levene tests of the homogeneity of variances were conducted to verify the assumptions of the general linear

model. The tests suggested that the variance of mean rating change significantly differed between the moderate negative disagreement and the strong positive disagreement conditions (p = .004), and between the moderate negative disagreement and the moderate positive disagreement conditions (p = .04). These results are likely due to the great sensitivity of these tests to trivial deviations from normality which are often found in large samples. Other possible comparisons showed homogeneous variances between conditions (p range = .06 – .75). A visual inspection of our outcome measure using *P-P* plots suggested that the distribution of mean rating change observed in each type of group disagreement approximately matched the theoretical normal distribution. This was also supported by relatively low ranges of skewness and kurtosis parameters (*skewness*: -0.001 – 0.16; *kurtosis*: -0.09 – 0.60).

An inter-item reliability analysis performed on the item scores collected on the 262 pre-registered study participants showed that both childhood and current unpredictability and harshness questionnaires had a satisfactory internal consistency (*Cronbach's alphas*: *childhood harshness* = .83, *childhood unpredictability* = .83 ; *current harshness* = .91, *current unpredictability* = .86). The two childhood environment questionnaires were moderately correlated (r = .41), so were the two current environment questionnaires (r = .45). The childhood and current harshness questionnaires were weakly correlated (r = .16), so were the childhood and current unpredictability questionnaires (r = .24). None of the participants were outliers on childhood/current harshness and childhood/current unpredictability scores.

2.4.3. Distributions of mean rating change in each type experimental condition

The Supplementary Figure S9 shows the distributions of mean rating change in each experimental condition (moderate/negative disagreement, strong/negative disagreement, moderate/positive disagreement, strong/positive disagreement, agreement, no feedback).

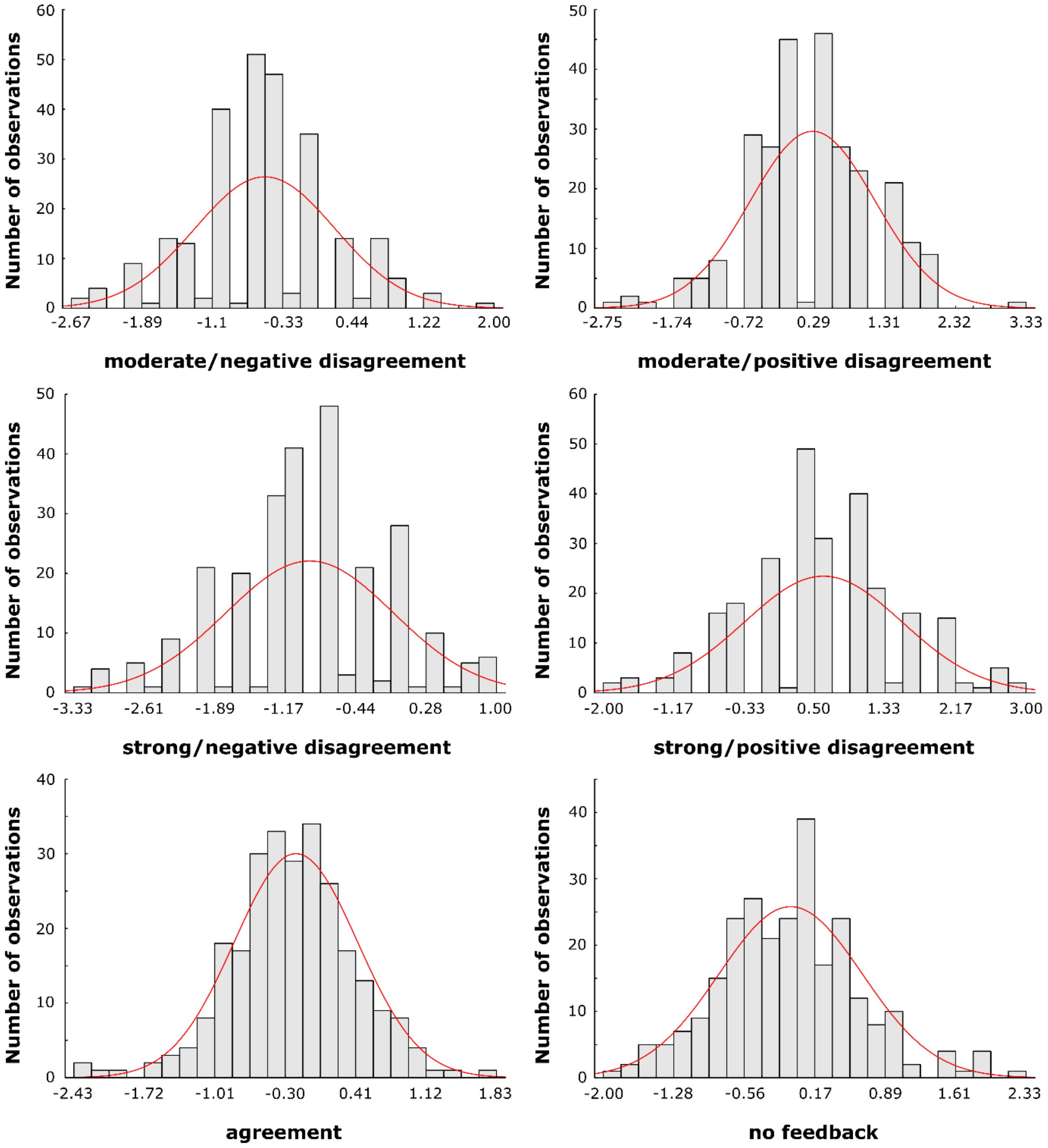

Supplementary Figure S9. Distributions of mean rating change in each experimental condition

2.4.4. Distributions of scores in environmental variables and their relation with residuals

The Supplementary Figure S10 shows the distributions of childhood/current harshness and unpredictability scores as well as their relation with residuals.

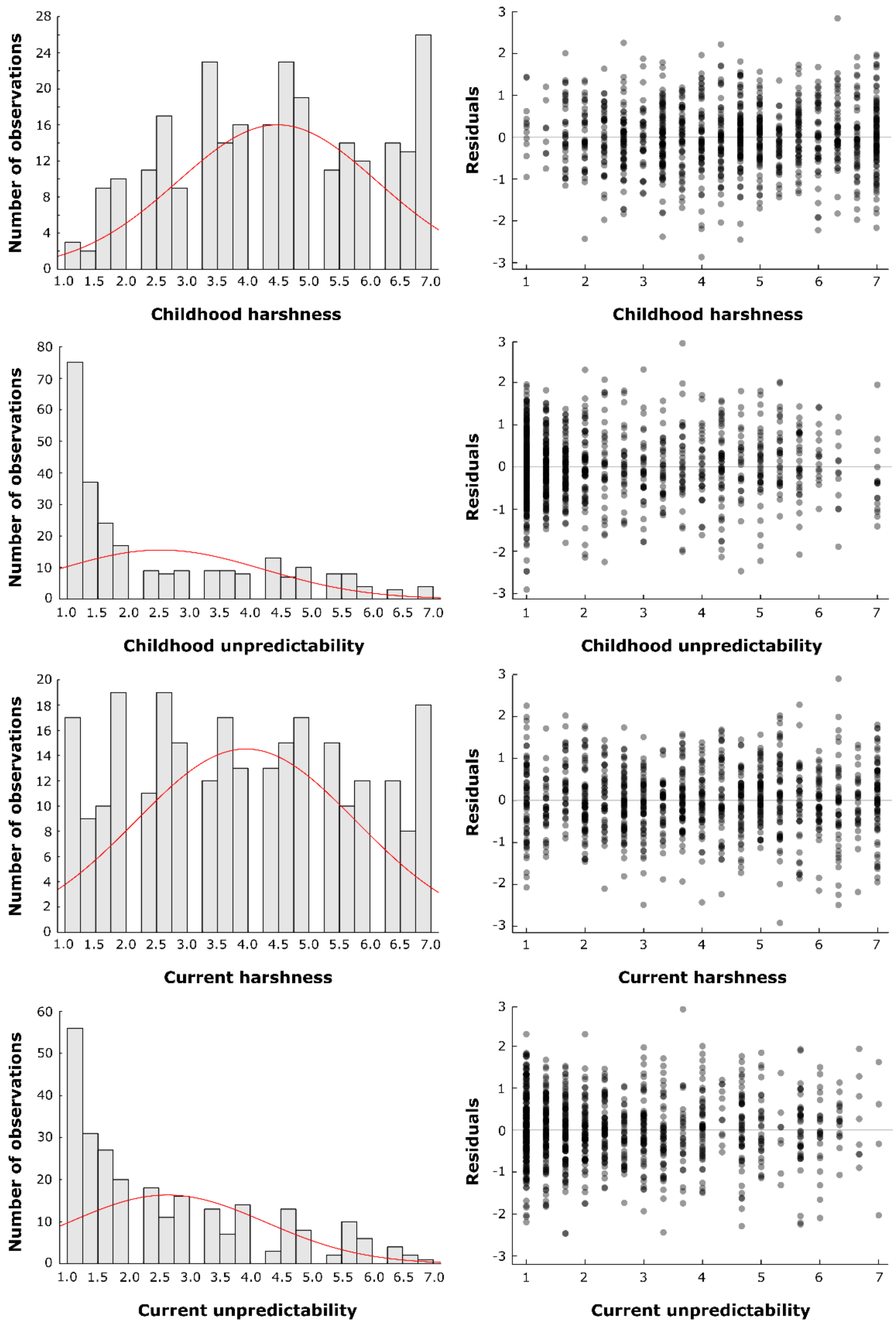

Supplementary Figure S10. Distributions of scores in environmental variables and their relation with residuals.

2.4.5. Correlation of mean rating change and environment variables for each type of disagreement trials

The Supplementary Figure S11 (childhood environment) and S12 (current environment) show the correlations of participants' performance in terms of mean rating change and environment variables. The correlation matrices with *r* coefficients are inserted below (Supplementary Table S2 and S3).

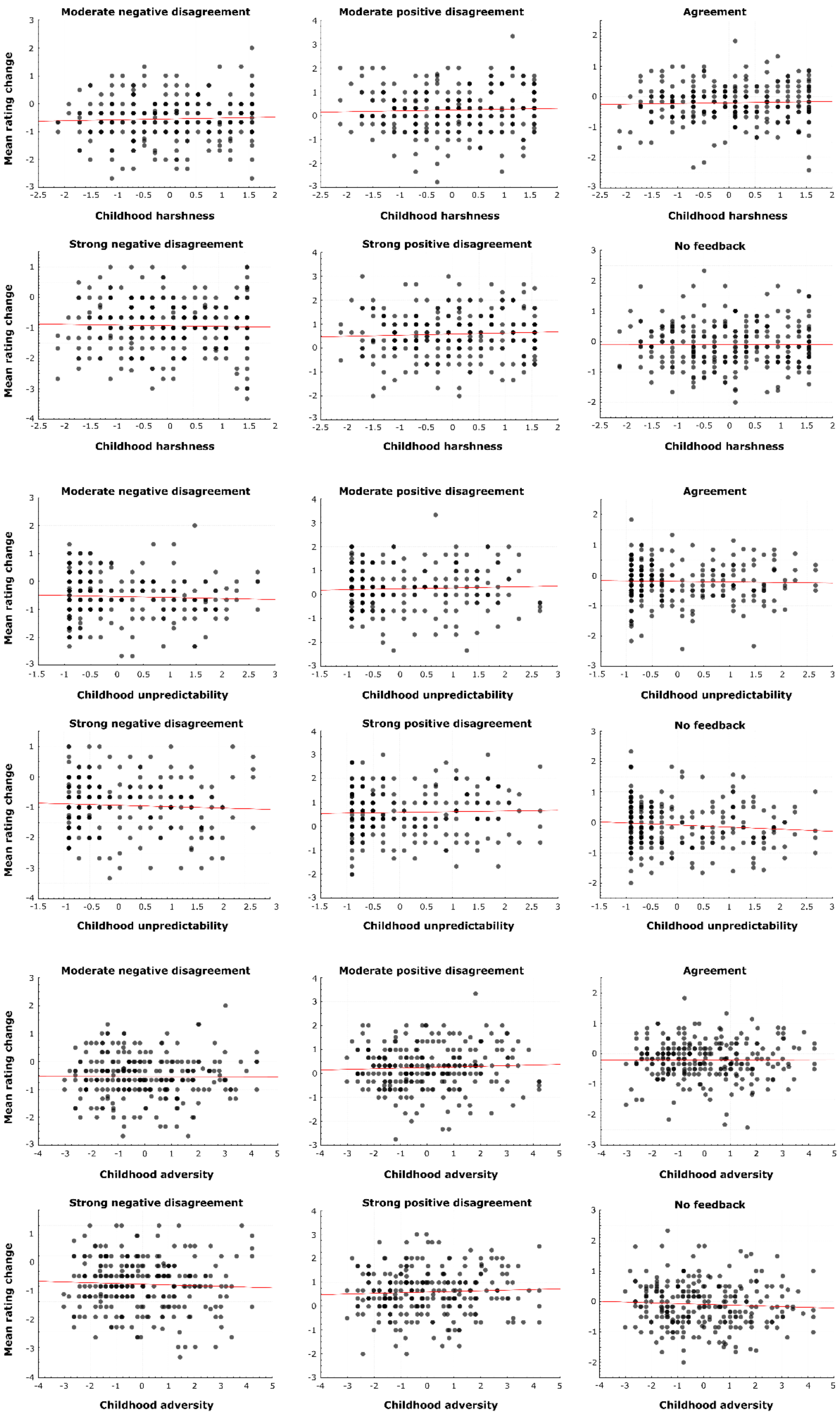

Supplementary Figure S11. Correlation of mean rating change obtained in each type of disagreement trials and each childhood environment variable.

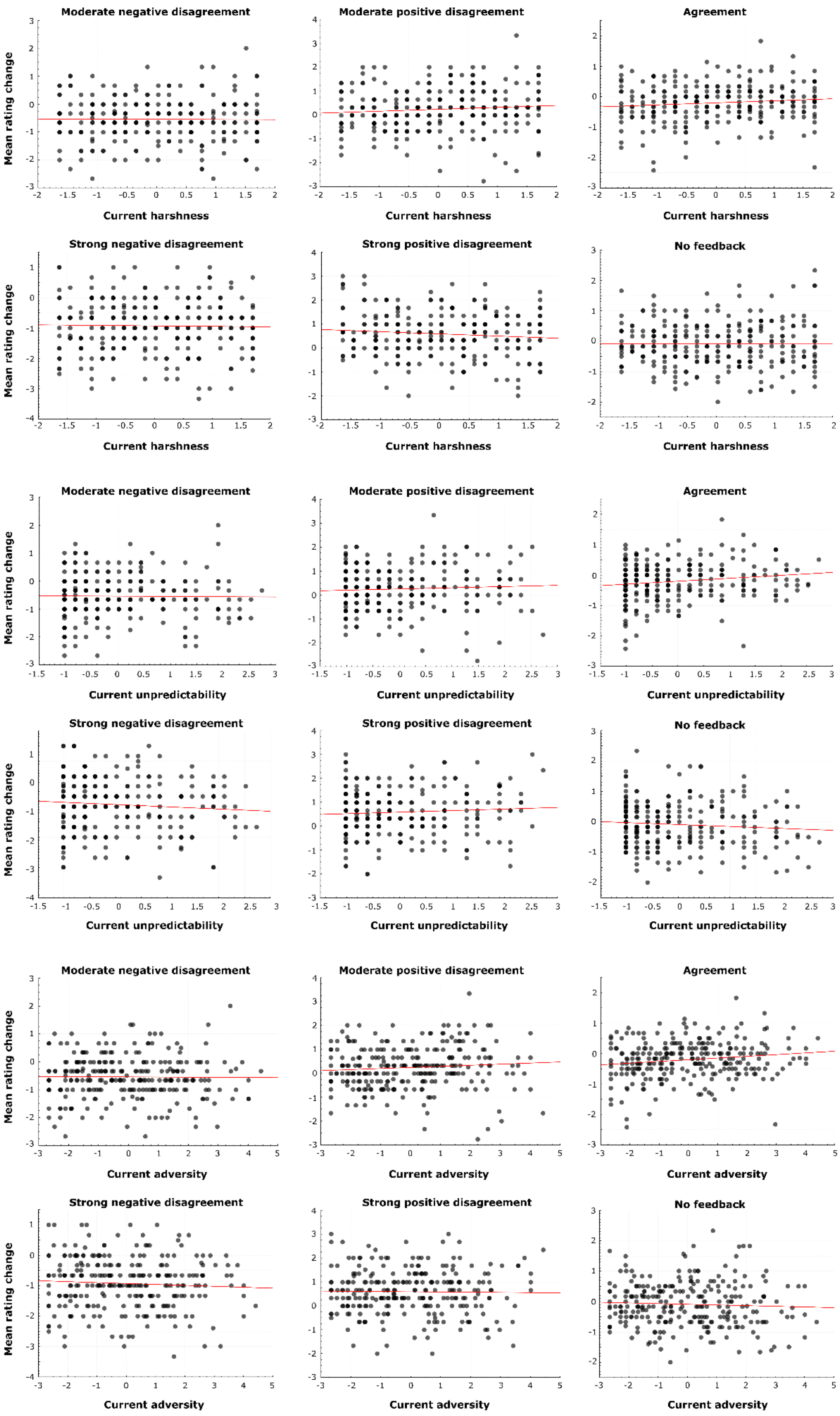

Supplementary Figure S12. Correlation of mean rating change obtained in each type of disagreement trials and each current environment variable.

2.4.6. Correlation matrices of mean rating change and environmental variables

The correlation matrices with *r* coefficients are inserted below (Supplementary Table S2 and S3).

				Childhood harshness	Childhood unpredictability	Childhood adversity
Mean rating change	Disagreement trials	negative	moderate	-0,04	0,05	0,00
			strong	-0,03	0,05	0,05
		positive	moderate	0,04	0,04	0,05
			strong	0,05	0,03	0,05
	Agreement trials			0,04	-0,03	0,00
	No feedback trials			-0,00	-0,09	-0,06

Supplementary Table S2. Correlation matrix – Childhood environment (*r* coefficients).

				Current harshness	Current unpredictability	Current adversity
Mean rating change	Disagreement trials	negative	moderate	0,01	0,01	0,01
			strong	0,02	0,09	0,06
		positive	moderate	0,09	0,06	0,09
			strong	-0,10	0,07	-0,02
	Agreement trials			0,11	0,16	0,15
	No feedback trials			-0,00	-0,09	-0,05

Supplementary Table S3. Correlation matrix – Current environment (*r* coefficients).

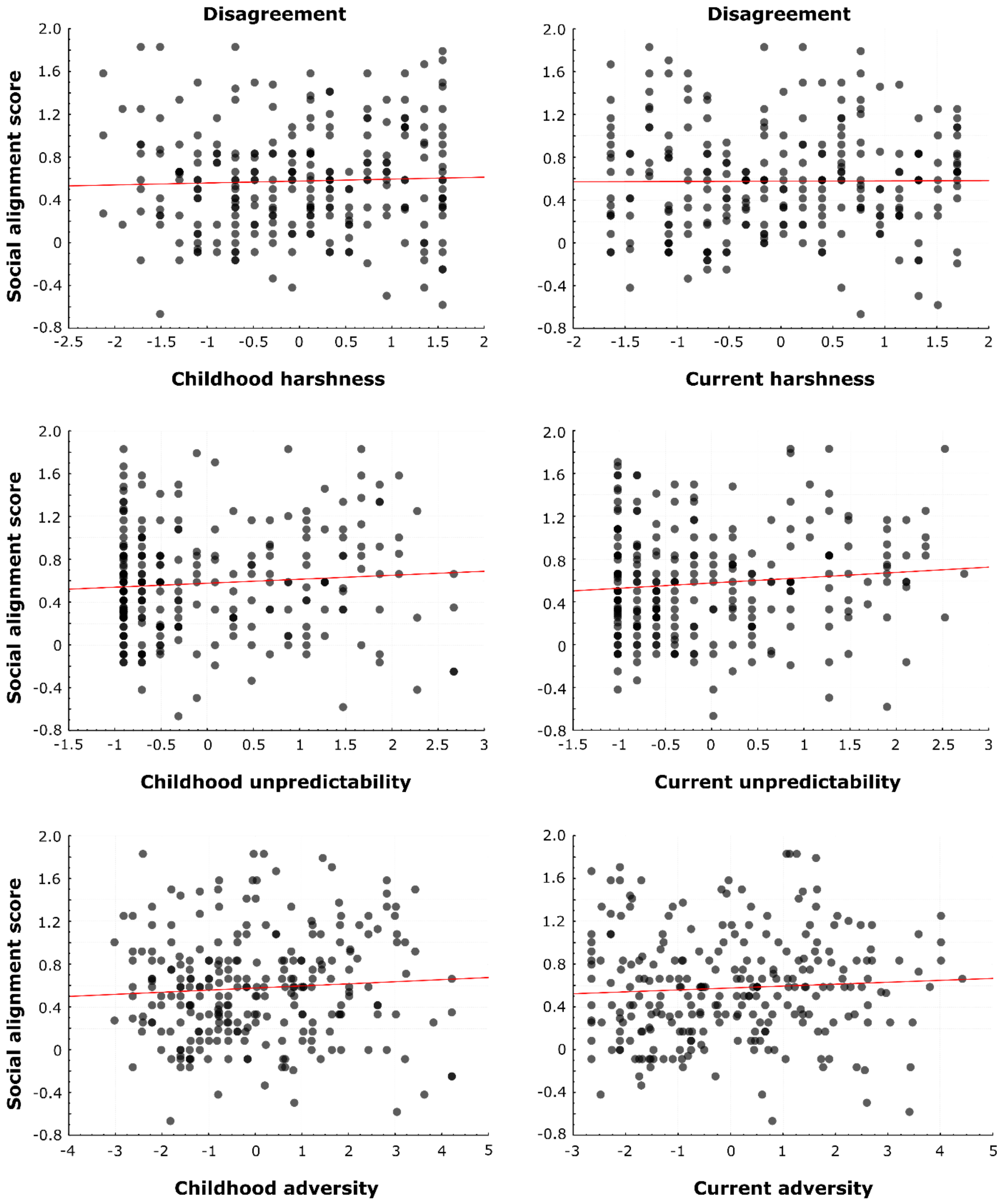

Supplementary Figure S13. Correlation of social alignment scores obtained in disagreement trials and environment variables (childhood and current).

2.4.7. Testing the adequacy of the computational model of choice

All the effects revealed by the analyses of the participants' social alignment scores were also obtained with the model's predictions (figure 4.b of the manuscript). Using *t*-tests for single mean and *t*-tests for dependent samples, we first showed that the model accurately predicts an increase in social alignment scores in the positive disagreement trials of moderate ($M = 0.46$, $SEM = 0.02$, $t(261) = 19.25$, $p < .001$) and strong amplitude ($M = 0.70$ $SEM = 0.04$, $t(261) = 19.53$, $p < .001$), and in the negative disagreement trials of moderate ($M = 0.48$, $SEM = 0.02$, $t(261) = 19.69$, $p < .001$) and strong amplitude ($M = 0.72$, $SEM = 0.04$, $t(261) = 19.81$, $p < .001$) (figure 4.b of the manuscript).

A linear mixed model ran on model-predicted social alignment scores, and including disagreement valence and disagreement strength as fixed factors, and participants' ID as a random factor, showed a pattern of results that partly reproduced the pattern found in the equivalent model ran on real scores. The computational model predicted a main effect of the disagreement strength factor, with greater scores in the strong than moderate disagreement trials ($\beta = 0.24 \pm 0.01$, $t(783) = 20.29$, $p < .001$). However, it did not predict the main effect of disagreement valence ($\beta = -0.02 \pm 0.01$, $t(783) = -1.38$, $p = .17$) observed on the real dataset, and that was characterized by a greater alignment score in negative disagreement than in positive disagreement. As in the real data, the model predicted an absence of interaction between disagreement valence and disagreement strength on social alignment scores ($\beta = -0.002 \pm 0.02$, $t(783) = 0.12$, $p = .90$).

Finally, the model-predicted scores explained a significant component of scores calculated from behavioural data (moderate positive disagreement: adjusted $R^2 = .34$, $df = 260$, $F = 133.14$, p < .001; strong positive disagreement: adjusted $R^2 = .39$, $df = 260$, $F = 166.37$, p < .001; moderate negative disagreement: adjusted $R^2 = .18$, $df = 260$, $F = 56.37$, p < .001; strong negative disagreement: adjusted $R^2 = .49$, $df = 260$, $F = 253.50$, p < .001; Supplementary Figure S14.ab).

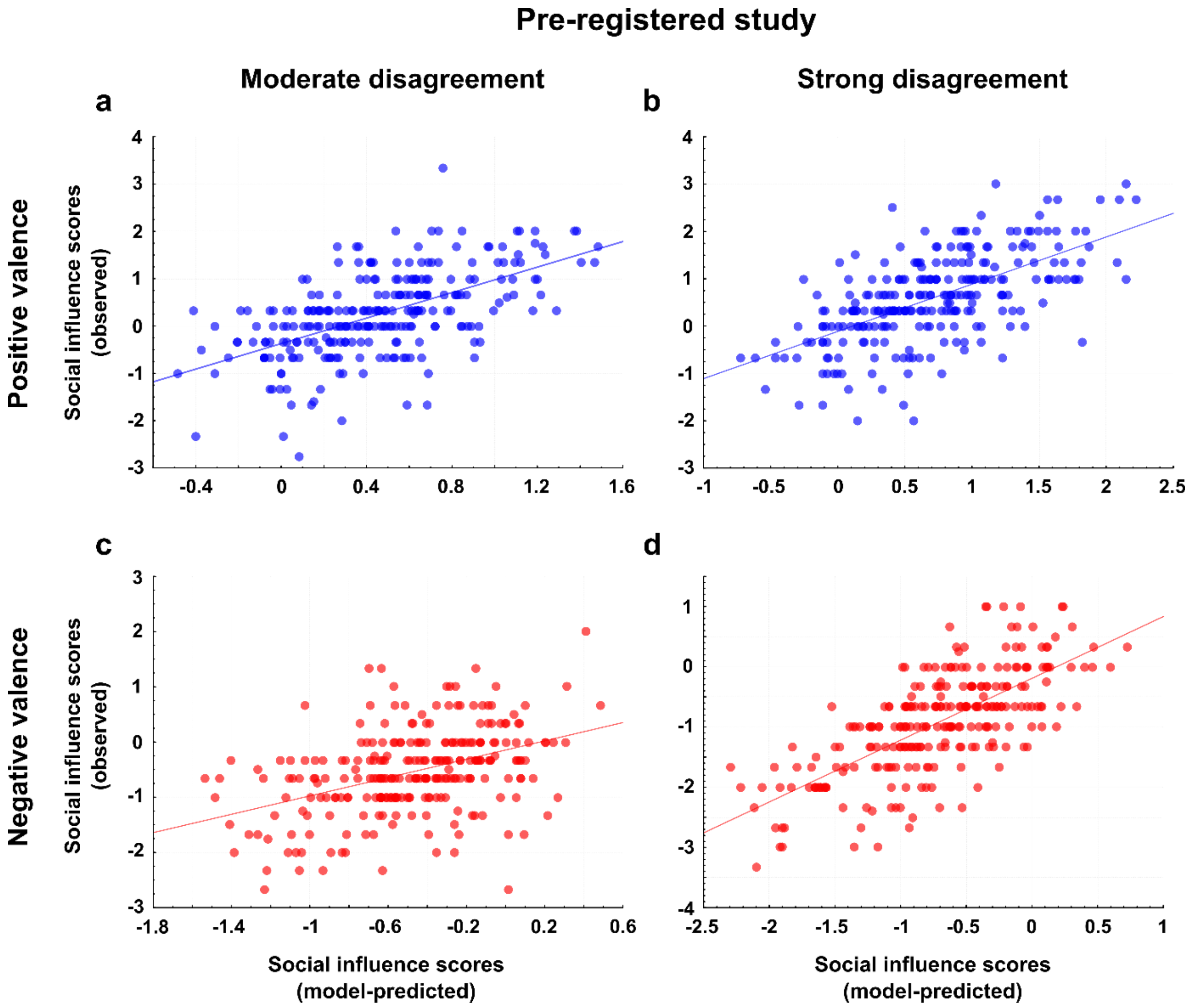

Supplementary Figure S14. Pre-registered study. Observed adjustment scores regressed on model-predicted social alignment scores. Positive disagreement of **a.** moderate and **b.** strong magnitude. Negative disagreement of **c.** moderate and **d.** strong magnitude.

2.5. Unregistered analyses

2.5.1. Slope outliers detection

Slope outliers in each regression (i.e., the main effect of childhood harshness, childhood unpredictability, current harshness, and current unpredictability on social alignment scores following disagreements) were identified by calculating each participants' Cook's Distance, and were excluded from unregistered analyses if the data point's Cook's value was greater than a standard cut-off of $4/N_{\text{participants}}$ [9] (see Supplementary Figure S15). In total, 14

additional participants were removed from the pre-registered dataset, which represents 5% of the original sample.

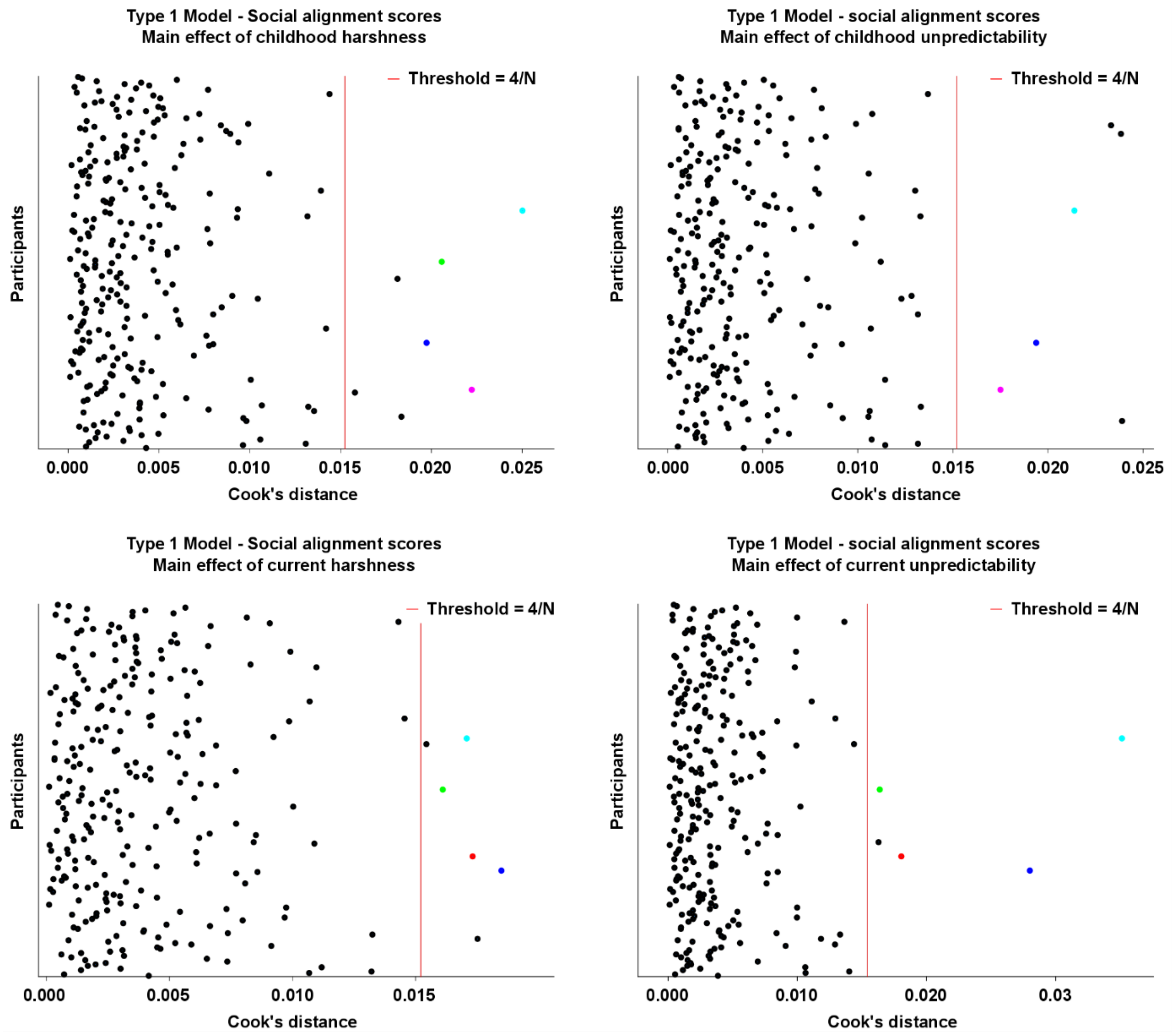

Supplementary Figure S15. Slope outliers detection. Each panel represents the Cook's distance (x axis) of each participants (y axis, black dots) calculated from each type 1 model which included an environmental variable as the main predictor of social alignment scores. The cut-off (red vertical bars) is set at 4/(*N*=262) following standard methods [9]. Participants whose Cook's distance were beyond this threshold were excluded from the unregistered analyses. Colored dots are participants who had a Cook's distance beyond the threshold in more than one regression.

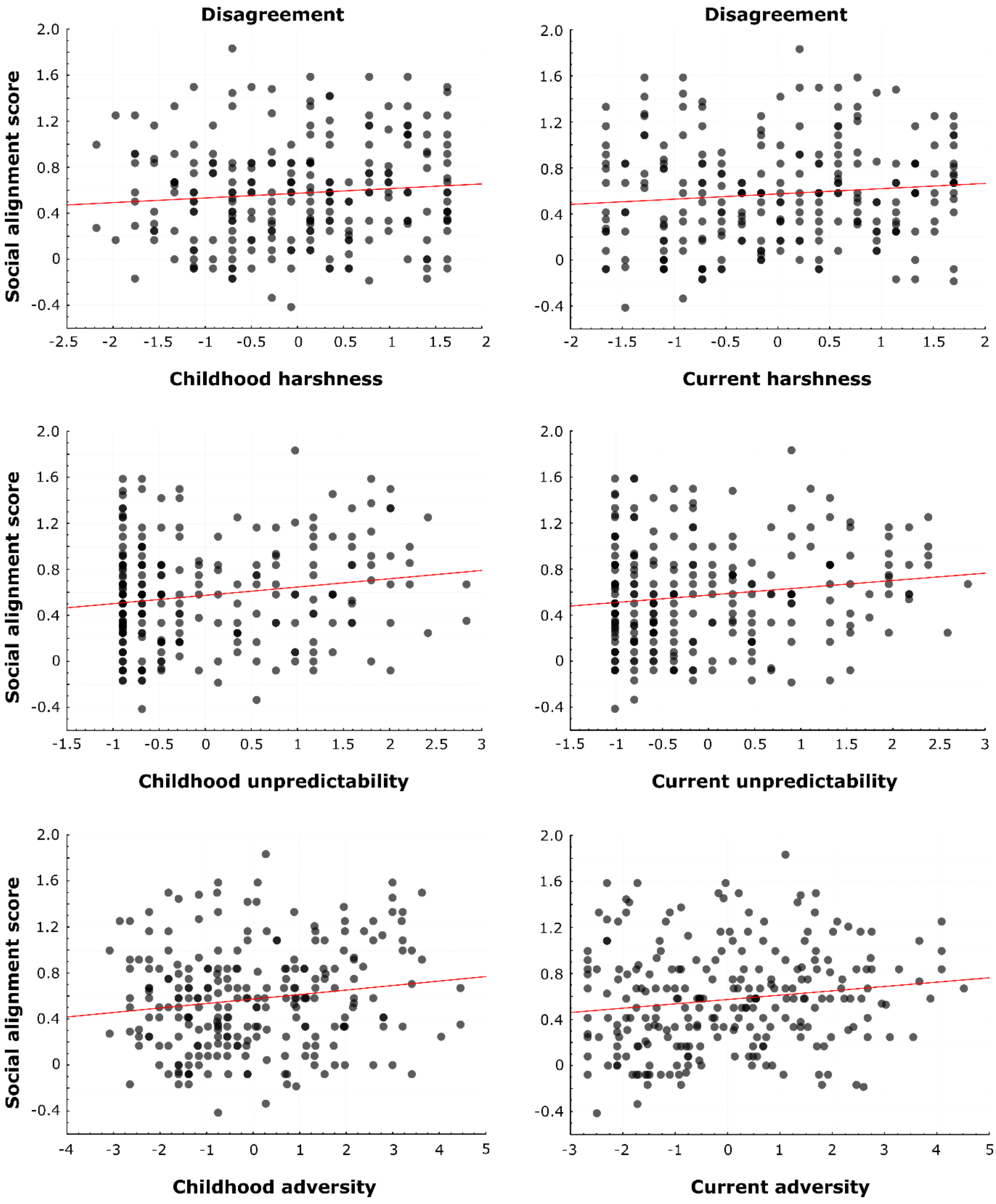

Supplementary Figure S16. Pre-registered study – slope outliers removed. Correlation of social alignment scores obtained in disagreement trials and environment variables (childhood and current).

References

1. Todorov A, Duchaine B. 2008 Reading trustworthiness in faces without recognizing faces. Cogn. Neuropsychol. 25,395-410. (DOI: 10.1080/02643290802044996)
2. Griskevicius V, Delton AW, Robertson TE, Tybur JM. 2011 Environmental contingency in life history strategies: the influence of mortality and socioeconomic status on reproductive timing. J. Pers. Soc. Psychol. 100,241–254. (DOI: http://dx.doi.org/10.1037/a0021082)
3. Griskevicius V, Ackerman JM, Cantú SM, Delton AW, Robertson TE, Simpson JA, Tybur JM. 2013 When the economy falters, do people spend or save? Responses to resource scarcity depend on childhood environments. Psychol. Sci. 24,197–205. (DOI: https://doi.org/10.1177/0956797612451471)
4. Mittal C, Griskevicius V. 2014 Sense of control under uncertainty depends on people's childhood environment: A life history theory approach. J. Pers. Soc. Psychol. 107,621-637. (DOI: http://dx.doi.org/10.1037/a0037398)
5. Mittal C, Griskevicius V, Simpson JA, Sung S, Young ES. 2015 Cognitive Adaptations to Stressful Environments: When Childhood Adversity Enhances Adult Executive Function. J. Pers. Soc. Psychol. 109,604–621. (DOI: 10.1037/pspi0000028.)
6. Hill SE, Prokosch ML, DelPriore DJ, Griskevicius V, Kramer A. 2016 Low Childhood Socioeconomic Status Promotes Eating in the Absence of Energy Need. Psychol. Sci. 27,354-364. (DOI: 10.1177/0956797615621901)
7. Mell H, Safra L, Algan Y, Baumard N, Chevallier C. 2017 Childhood environmental harshness predicts coordinated health and reproductive strategies: A cross-sectional study of a nationally representative sample from France. Evol. Hum. Behav. In press. (DOI: http://dx.doi.org/10.1016/j.evolhumbehav.2017.08.006)

8. Faul F, Erdfelder E, Lang A, Buchner A. 2007 GPower3: a flexible statistical power analysis program for the social, behavioral, and biomedical sciences. Behav. Res. Methods 39,175–191. (DOI: 10.3758/BF03193146)

9. Bollen KA, Jackman RW. 1985 Regression diagnostics: an expository treatment of outliers and influential cases. Sociol. Methods Res. 13,510–542.
